# Diagnostic Performance of RO948 F 18 Tau Positron Emission Tomography in the Differentiation of Alzheimer Disease From Other Neurodegenerative Disorders

**DOI:** 10.1001/jamaneurol.2020.0989

**Published:** 2020-05-11

**Authors:** Antoine Leuzy, Ruben Smith, Rik Ossenkoppele, Alexander Santillo, Edilio Borroni, Gregory Klein, Tomas Ohlsson, Jonas Jögi, Sebastian Palmqvist, Niklas Mattsson-Carlgren, Olof Strandberg, Erik Stomrud, Oskar Hansson

**Affiliations:** 1 Clinical Memory Research Unit, Department of Clinical Sciences, Lund University, Malmö, Sweden; 2 Department of Neurology, Skåne University Hospital, Lund, Sweden; 3 Alzheimer Center Amsterdam, Department of Neurology, Amsterdam Neuroscience, Amsterdam University Medical Center, Amsterdam, the Netherlands; 4 Memory Clinic, Skåne University Hospital, Malmö, Sweden; 5 F. Hoffmann-La Roche Ltd, Basel, Switzerland; 6 Department of Radiation Physics, Skåne University Hospital, Lund, Sweden; 7 Skåne University Hospital, Department of Clinical Physiology and Nuclear Medicine, Lund, Sweden; 8 Wallenberg Centre for Molecular Medicine, Lund University, Lund, Sweden

## Abstract

**Importance:**

The diagnostic performance of second-generation tau positron emission tomographic (PET) tracers is not yet known.

**Objective:**

To examine the novel tau PET tracer RO948 F 18 ([^18^F]RO948) performance in discriminating Alzheimer disease (AD) from non-AD neurodegenerative disorders.

**Design, Setting, and Participants:**

In this diagnostic study, 613 participants in the Swedish BioFINDER-2 study were consecutively enrolled in a prospective cross-sectional study from September 4, 2017, to August 28, 2019. Participants included 257 cognitively unimpaired controls, 154 patients with mild cognitive impairment, 100 patients with AD dementia, and 102 with non-AD neurodegenerative disorders. Evaluation included a comparison of tau PET tracer [^18^F]RO948 with magnetic resonance imaging (MRI) and cerebrospinal fluid and a head-to-head comparison between [^18^F]RO948 and flortaucipir F 18 ([^18^F]flortaucipir) in patients with semantic variant primary progressive aphasia (svPPA).

**Exposures:**

[^18^F]RO948 (all patients) and [^18^F]flortaucipir (3 patients with svPPA) tau PET; MRI (hippocampal volume, composite temporal lobe cortical thickness, whole-brain cortical thickness) and cerebrospinal fluid measures (p-tau181 and amyloid Aβ42 and Aβ40 ratio[Aβ42/Aβ40], and Aβ42/p-tau181 ratio).

**Main Outcomes and Measures:**

Standard uptake value ratios (SUVRs) in 4 predefined regions of interest (ROIs) reflecting Braak staging scheme for tau pathology and encompass I-II (entorhinal cortex), III-IV (inferior/middle temporal, fusiform gyrus, parahippocampal cortex, and amygdala), I-IV, and V-VI (widespread neocortical areas), area under the receiver operating characteristic curve (AUC) values, and subtraction images between [^18^F]RO948 and [^18^F]flortaucipir.

**Results:**

Diagnostic groups among the 613 participants included cognitively unimpaired (mean [SD] age, 65.8 [12.1] years; 117 men [46%]), mild cognitive impairment (age, 70.8 [8.3] years; 82 men [53%]), AD dementia (age, 73.5 [6.7] years; 57 men [57%]), and non-AD disorders (age, 70.5 [8.6] years; 41 men [40%]). Retention of [^18^F]RO948 was higher in AD dementia compared with all other diagnostic groups. [^18^F]RO948 could distinguish patients with AD dementia from individuals without cognitive impairment and those with non-AD disorders, and the highest AUC was obtained using the I-IV ROI (AUC = 0.98; 95% CI, 0.96-0.99 for AD vs no cognitive impairment and AUC = 0.97; 95% CI, 0.95-0.99 for AD vs non-AD disorders), which outperformed MRI (highest AUC = 0.91 for AD vs no cognitive impairment using whole-brain thickness, and AUC = 0.80 for AD vs non-AD disorders using temporal lobe thickness) and cerebrospinal fluid measures (highest AUC = 0.94 for AD vs no cognitive impairment using Aβ42/p-tau181, and AUC = 0.93 for AD vs non-AD disorders using Aβ42/Aβ40). Generally, tau PET positivity using [^18^F]RO948 was observed only in Aβ-positive cases or in *MAPT* R406W mutation carriers. Retention of [^18^F]RO948 was not pronounced in patients with svPPA, and head-to-head comparison revealed lower temporal lobe uptake than with [^18^F]flortaucipir.

**Conclusions and Relevance:**

In this study, elevated [^18^F]RO948 SUVRs were most often seen among Aβ-positive cases, which suggests that [^18^F]RO948 has high specificity for AD-type tau and highlights its potential as a diagnostic marker in the differential diagnosis of AD.

## Key Points

**Question:**

How does RO948 F 18 positron emission tomographic scanning discriminate between Alzheimer disease and other neurodegenerative disorders in comparison with magnetic resonance imaging and cerebrospinal fluid measures?

**Findings:**

In this diagnostic study including 613 patients from the Swedish BioFINDER-2 clinical trial, standard uptake value ratios of RO948 F 18 were higher in patients with Alzheimer disease dementia compared with cognitively unimpaired controls and patients with other neurodegenerative disorders; furthermore, RO948 F 18 outperformed magnetic resonance imaging and cerebrospinal fluid measures. Generally, tau positron emission tomographic positivity was confined to amyloid β–positive cases or *MAPT* R406W mutation carriers in this cohort; in patients with semantic variant primary progressive aphasia, RO948 F 18 retention was lower than that for flortaucipir F 18.

**Meaning:**

These findings suggest that RO948 F 18 has a high specificity for Alzheimer disease–type tau and highlight its potential as a diagnostic marker in the workup of patients treated in memory clinics.

## Teaser

This diagnostic study examines the use of RO948 F 18 in tau positron emission tomographic imaging as a diagnostic marker for identification of Alzheimer disease compared with magnetic resonance imaging and cerebrospinal fluid measures.

## Introduction

Alzheimer disease (AD) is characterized by deposition of amyloid β (Aβ) in senile plaques and hyperphosphorylated tau in neurofibrillary tangles,^1^ which consist mainly of paired helical filaments (PHFs) comprising a mixture of 3- to 4-repeat tau isoforms.^2,3^ Tau pathology is also seen in various non-AD neurodegenerative disorders, including progressive supranuclear palsy and other variants of frontotemporal lobar degeneration.^4^ Tau deposits in these disorders, however, differ from the AD-type PHFs consisting of 3- to 4 repeat tau isoforms.^5^ In AD, cortical neurofibrillary neurotangles are closely associated with neurodegeneration and onset of cognitive impairment.^6^

Tau-selective positron emission tomographic (PET) tracers have facilitated in vivo studies of tau pathology in neurodegenerative disorders.^7^ The most commonly studied tau PET tracer, flortaucipir F 18 ([^18^F]flortaucipir),^8^ discriminates AD from other neurodegenerative diseases and outperforms magnetic resonance imaging (MRI).^9^ However, [^18^F]flortaucipir has off-target binding in the basal ganglia, brainstem, and choroid plexus^10^ and has elevated retention in the semantic variant of primary progressive aphasia (svPPA),^11^ a condition most often associated with TDP-43 type C pathology.^12,13^ Other tau PET tracers include GTP1 F 18,^14^ MK6240 F 18,^15,16^ and RO948 F 18([^18^F]RO948).^17^ While structurally similar to [^18^F]flortaucipir, [^18^F]RO948^18,19,20^ has more rapid kinetics and less off-target binding in the basal ganglia.^19,21^ The diagnostic performance of these second-generation tau PET tracers is not known. To our knowledge, there have been no head-to-head comparisons with other imaging or cerebrospinal fluid (CSF) markers. We tested [^18^F]RO948 tau PET for discriminating AD from non-AD in patients with neurodegenerative disorders, in individuals with no cognitive impairment, and in comparison with established MRI and CSF markers. We also directly compared [^18^F]RO948 and [^18^F]flortaucipir in a subset of patients with svPPA to examine the specificity of both tracers.

## Methods

### Participants

In this diagnostic study, we included participants in the prospective and longitudinal Swedish BioFINDER-2 study, including individuals without cognitive impairment and patients with mild cognitive impairment (MCI), AD dementia, and non-AD neurodegenerative disorders.^22^ eMethods 1 in the Supplement presents the inclusion and exclusion criteria. Groups were established without the use of biomarkers, but cognitively unimpaired and MCI participants were subdivided based on Aβ status (CSF, Aβ42/Aβ40; cutoff <0.089, as defined in clinical practice at the Sahlgrenska University Hospital, Mölndal, Sweden). We included only patients with Aβ-positive AD dementia in accordance with the National Institute on Aging-Alzheimer Association research framework.^23^ In BioFINDER-2, Aβ PET is by design performed only in individuals without cognitive impairment and those with MCI; CSF Aβ42/Aβ40 was thus chosen to have a common measure of Aβ pathology across all enrollees. All participants gave written informed consent; no financial compensation was provided. Ethical approval was given by the Regional Ethical Committee in Lund, Sweden. Approval for PET imaging was obtained from the Swedish Medicines and Products Agency and the local radiation safety committee at Skåne University Hospital, Lund, Sweden. The present study was conducted from September 4, 2017, to August 28, 2019.

### Image Acquisition and Processing

Full PET details are described elsewhere^24^ and included in eMethods 2 in the Supplement. PET using [^18^F]RO948 was performed on a digital scanner (Discovery MI; GE Healthcare) 70 to 90 minutes following injection (list-mode acquisition). This protocol was chosen over full-dynamic scanning to make PET scanning feasible for the large number of patients recruited into BioFINDER-2, with the acquisition window selected using previous [^18^F]RO948 work.^19^ In the patients with svPPA who underwent an additional scan with [^18^F]flortaucipir, data were acquired 80 to 100 minutes after injection using the same digital scanner.^25^ Standardized uptake value ratio (SUVR) images were created using the inferior cerebellar cortex as the reference region.^26^ We report findings uncorrected for partial volume error or corrected (geometric transfer matrix)^27^ (eTable 3, eTable 4, eFigure 5, and eFigure 6 in the Supplement). A high-resolution T1-weighted MRI was acquired (3T MAGNETOM Prisma; Siemens Healthineers) for PET image coregistration and template normalization.

### Region-of-Interest Definition

To cover brain areas affected by neurofibrillary tangle pathology across the course of AD,^28,29^ 4 FreeSurfer-based composite regions of interest (ROIs) were created. These ROIs were originally developed using [^18^F]flortaucipir^28^ to approximate the Braak staging scheme for tau pathology^29^ and encompass I-II (entorhinal cortex), III-IV (inferior/middle temporal, fusiform gyrus, parahippocampal cortex, and amygdala), I-IV,^30,31^ and V-VI (widespread neocortical areas) (eFigure 1 in the Supplement). Individual tau-imaging ROIs (ie, I-II, III, IV, V, and VI) were also investigated. To examine the [^18^F]RO948 signal in regions that are comparatively unaffected by neurofibrillary tangle pathology,^29^ primary sensory and motor cortex ROIs were included. For the head-to-head comparison between [^18^F]RO948 and [^18^F]flortaucipir in svPPA, owing to the focal nature of retention with [^18^F]flortaucipir in patients with svPPA (confined to the anterior temporal lobe, including the white matter), participant-specific ROIs were drawn encompassing voxels showing elevated SUVRs within the temporal lobes, including subcortical white matter (eMethods 3 in the Supplement).

### Comparative Diagnostic Performance of [^18^F]RO948

For comparison with the [^18^F]RO948 SUVR, 3 predefined MRI markers were selected^9^: hippocampal volume (adjusted for intracranial volume), cortical thickness within a temporal meta-ROI encompassing regions susceptible to AD (mean thickness in the bilateral entorhinal, inferior/middle temporal, and fusiform cortices),^32^ and whole-brain cortical thickness. Furthermore, 3 CSF measures were included: p-tau181, Aβ42/Aβ40,^33^ and Aβ42/p-tau181.^34^ Cerebrospinal fluid p-tau181, Aβ42, and Aβ40 were quantified using enzyme-linked immunosorbent assays (Innotest, Fujirebio). Additional details can be found in eMethods 4 in the Supplement.

### Statistical Analysis

Groups were compared using Kruskal-Wallis or Fisher exact tests. Thresholds for tau PET positivity within composite ROIs were defined using the mean SUVR within a given ROI plus 2.5 SDs among 17 Aβ-negative young controls aged 20 to 40 years (eTable 1 in the Supplement).^35^ The cutoffs were chosen to slightly favor specificity for AD pathology over sensitivity. Mean [^18^F]RO948 SUVR images and scatterplots for ROIs are shown in eFigure 2 in the Supplement for these participants. Group differences in the [^18^F]RO948 SUVR were compared across ROIs (analysis of variance and post hoc *t* tests) and voxelwise (pairwise *t* tests, *P* < .001, *k*≥100) using SPM, version 12 software (eFigure 3 in the Supplement). Familywise error–corrected maps are shown in eFigure 4 in the Supplement. Owing to the small number of patients with svPPA, [^18^F]RO948 and [^18^F]flortaucipir were compared using a voxelwise subtraction analysis as a complement to the ROI-based comparison. Receiver operating characteristic analyses were performed to generate area under the curve (AUC) values (AD dementia and Aβ-positive MCI vs no cognitive impairment and non-AD). Differences in AUCs between modalities (PET, MRI, and CSF) were evaluated using bootstrapping (n = 1000).^36,37,38^ In addition to AUC, sensitivity and specificity, likelihood ratios (positive and negative), and percentage of agreement between classifiers (ie, diagnosis and [^18^F]RO948 SUVR: true outcomes and all outcomes) are reported. All analyses were performed in R, version 3.5.3 (R Foundation). Significance was set at a 2-sided level of *P* < .05.

## Results

### Participants

A total of 613 participants, including 257 cognitively unimpaired controls (38% Aβ-positive), 154 with MCI (62% Aβ-positive), 100 with AD dementia (100% Aβ-positive), and 102 with a non-AD neurodegenerative disorder (41% Aβ-positive; behavioral variant frontotemporal dementia [n = 12], svPPA [n = 7], dementia with Lewy bodies [n = 25], progressive supranuclear palsy [n = 16], Parkinson disease with or without cognitive impairment [n = 26]), multiple system atrophy [n = 6], and vascular dementia [n = 10]) were included. Baseline characteristics are provided in Table 1 and in the eResults and eTable 2 in the Supplement. Partial volume error–corrected [^18^F]RO948 PET SUVR data are presented in eTable 3 and eTable 4 in the Supplement. Regarding the high percentage of *APOE* ε4 carriership among controls, the BioFINDER-2 study, by design, enrolls younger (age, 40-65 years) and older (age, 65-100 years) controls, with the aim to build 2 study populations with 50% *APOE* ε4 carriers in each. This design was intended to enrich for Aβ pathology to be able to study the very early (preclinical) stages of AD. Details on both [^18^F]RO948 and [^18^F]flortaucipir in patients with svPPA are included in eTable 5 in the Supplement.

**Table 1.** Participant Characteristics

Characteristic	Cognitively unimpaired controls (n = 257)^a^	MCI (n = 154)	AD dementia (n = 100)	Non-AD (n = 102)^b^
Age, mean (SD) [range], y	65.8 (12.1) [41-89]	70.8 (8.3) [47-94]^c^	73.5 (6.7) [66-87]^c^^,^^d^^,^^e^	70.5 (8.6) [36-87]^c^
Sex, No. (%)				
Men	117 (46)	82 (53)	57 (57)	41 (40)^f^^,^^g^
Women	140 (54)	72 (47)	43 (43)	61 (60)
Education, mean (SD), y	12.7 (3.4)	12.3 (3.5)	12.2 (3.6)	12.4 (3.5)
MMSE score, mean (SD)	29 (1.15)^h^^,^^i^^,^^j^	27.1 (2.1)^h^^,^^j^	20 (4.3)	25 (4.6)^h^
Aβ status, No. (%)				
Negative	159 (62)	58 (38)	0	60 (59)
Positive	98 (38)	96 (62)	100 (100)	42 (41)
*APOE* ε4 status, No. (%)				
Negative	136 (53)	73 (47)	32 (32)	68 (67)
Positive	121 (47)	81 (53)^d^	68 (68)	34 (33)
RO948 F 18 SUVR within tau-imaging ROIs, mean (SD)^k^				
Braak stage I-II	1.16 (0.22)	1.36 (0.34)^e^^,^^l^	2.02 (0.40)^c^^,^^i^^,^^j^	1.21 (0.26)
Braak stage III-IV	1.17 (0.15)	1.30 (0.32)^e^^,^^l^	2.18 (0.70)^c^^,^^i^^,^^j^	1.20 (0.17)
Braak stage I-IV	1.17 (0.15)	1.31 (0.30)^e^^,^^l^	2.19 (0.58)^c^^,^^i^^,^^j^	1.19 (0.15)
Braak stage V-VI	1.06 (0.10)	1.08 (0.16)	1.53 (0.21)^c^^,^^i^^,^^j^	1.05 (0.11)

Abbreviations: Aβ, amyloid β; AD, Alzheimer disease; MCI, mild cognitive impairment; MMSE; Mini-Mental State Examination; ROI, region of interest; SUVR, standard uptake value ratio.

^a^ Regarding the high percentage of *APOE* ε4 carriership among controls, by design, the BioFINDER-2 study enrolls younger (age, 40-65 years) and older (age, 65-100 years) controls, with the aim to build 2 study populations with 50% *APOE* ε4 carriers in each. This design was intended to enrich for Aβ pathologic characteristics to be able to study the very early (preclinical) stages of AD.

^b^ Neurodegenerative disorders other than AD.

^c^ Significantly higher than cognitively unimpaired controls, *P* < .001.

^d^ Significantly higher than MCI, *P* < .05.

^e^ Significantly higher than non-AD, *P* < .01.

^f^ Significantly higher than AD dementia, *P* < .05.

^g^ Significantly higher than cognitively unimpaired controls, *P* < .05.

^h^ Significantly higher than AD dementia, *P* < .001.

^i^ Significantly higher than MCI, *P* < .001.

^j^ Significantly higher than non-AD, *P* < .001.

^k^ Braak stage I-II, entorhinal cortex; III-IV, inferior/middle temporal, fusiform gyrus, parahippocampal cortex, and amygdala; I-IV; and V-VI, widespread neocortical.

^l^ Significantly higher than cognitively unimpaired controls, *P* < .01.

### [^18^F]RO948 SUVRs Across Tau-Imaging Regions

**Figure 1.**
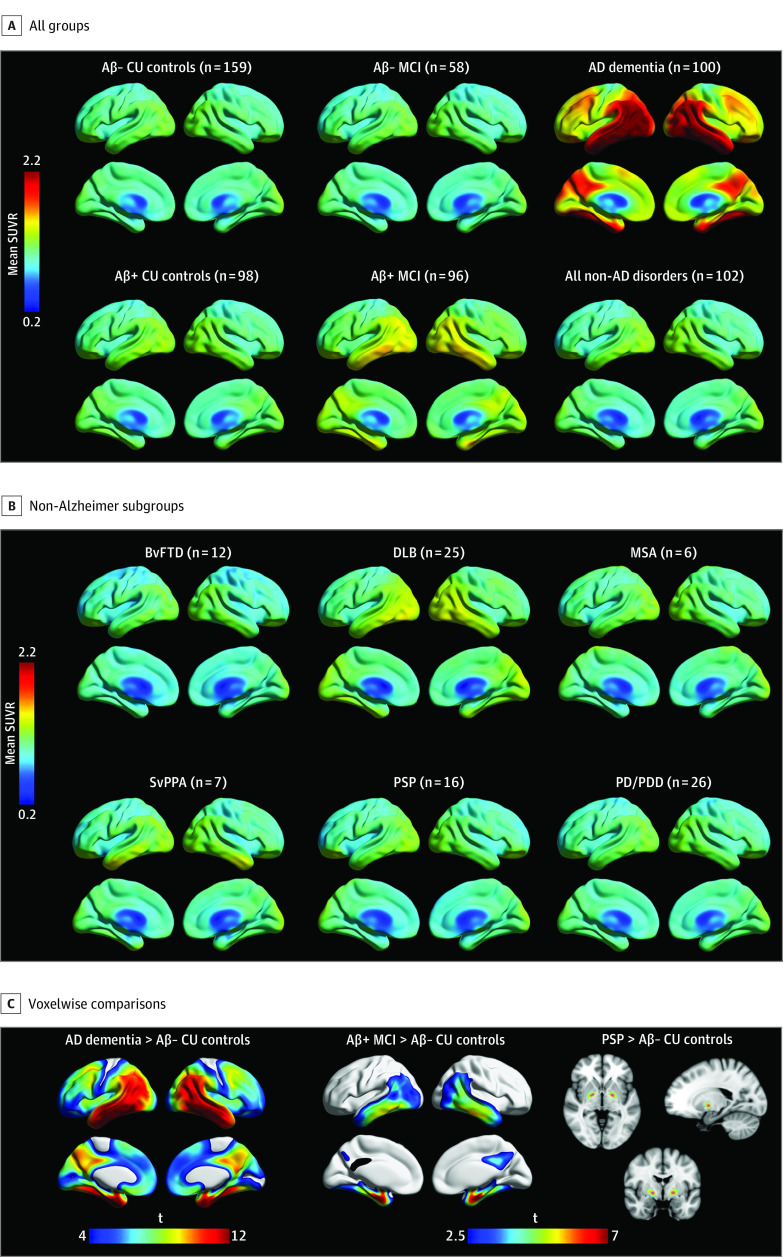
Mean RO948 F 18 ([^18^F]RO948) Standardized Uptake Value Ratio (SUVR) Images Across Diagnostic Groups A, Cognitively unimpaired (CU) control individuals and patients with mild cognitive impairment (MCI) and Alzheimer disease (AD) dementia. AD dementia was greater than amyloid β (Aβ)-negative CU controls and Aβ-positive MCI. B, Non-AD subgroups compared with Aβ-negative CU controls. Aβ vs *APOE* ε4 status (% positive) by non-AD subgroup was as follows: behavioral variant of frontotemporal dementia (BvFTD) (50% vs 33%), dementia with Lewy bodies (DLB) (60% vs 52%), multiple system atrophy (MSA) (33% vs 33%), semantic variant primary progressive aphasia (svPPA) (71% vs 17%), progressive supranuclear palsy (PSP) (38% vs 31%), and Parkinson disease/Parkinson disease dementia (PD/PDD) (23% vs 27%). C, Voxelwise group differences in [^18^F]RO948 SUVRs. AD dementia, Aβ-positive MCI, and PSP are compared with Aβ-negative CU controls. A cluster threshold of 100 voxels was applied with no correction for multiple comparisons (*P* < .001). t indicates t value.

**Figure 2.**
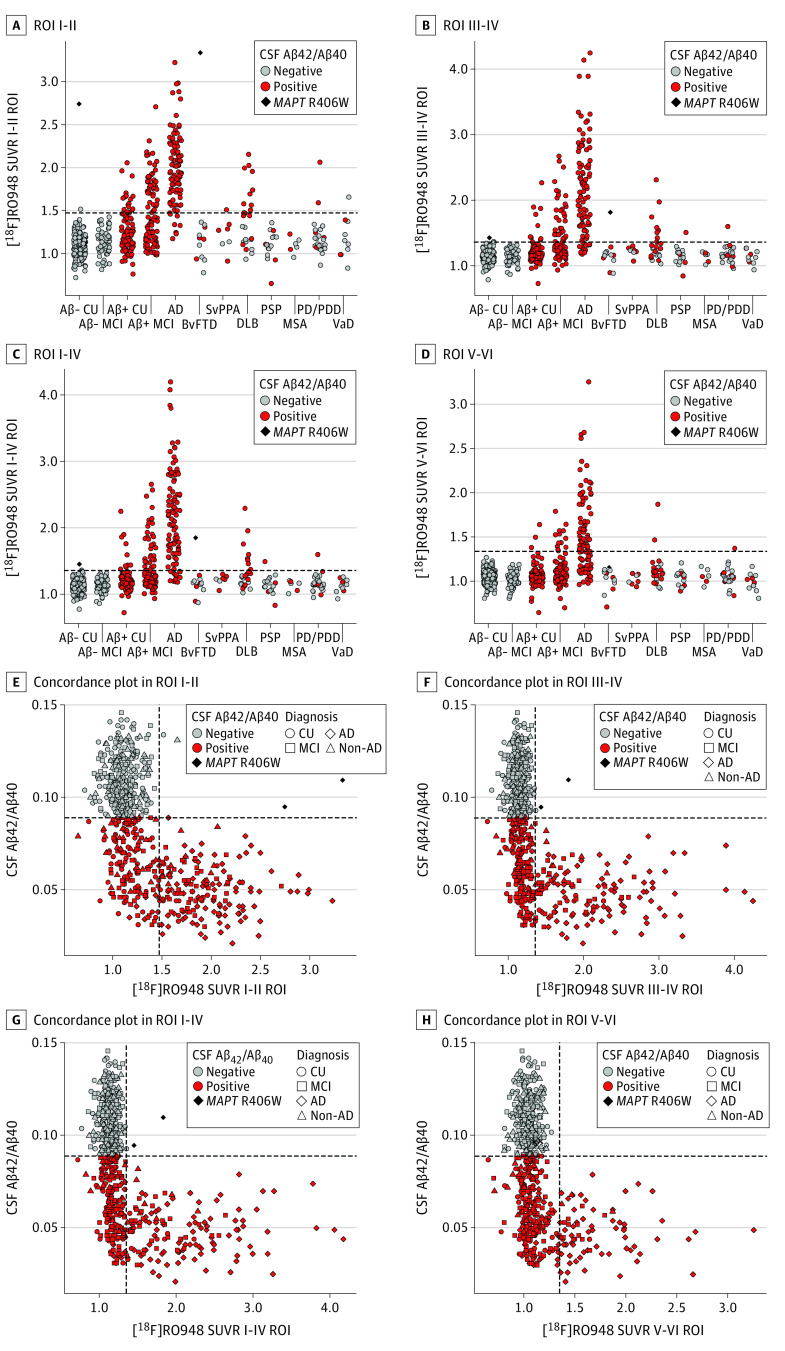
Mean RO948 F 18 ([^18^F]RO948) Standardized Uptake Value Ratios (SUVRs) Across Diagnostic Groups Within Tau-Imaging Regions of Interest (ROIs) ROI groupings were I-II (A), III-IV (B), I-IV (C), and V-VI (D). Concordance plots are shown between the [^18^F]RO948 SUVR and cerebrospinal fluid (CSF) amyloid β_42_ and β_40_ (Aβ42/Aβ40) for ROIs I-II (E), III-IV (F), I-IV (G), and V-VI (H). The horizontal dashed lines indicate the cutoffs for tau positivity across the ROIs, defined using the mean plus 2.5 SDs in Aβ-negative young controls (I-II ROI>1.48; III-IV and I-IV ROIs >1.36; V-VI ROI>1.35). The vertical dashed line indicates the cutoff for Aβ-positivity (CSF Aβ42/Aβ40<0.089, as established in an independent population by the neurochemistry laboratory at the Sahlgrenska University Hospital, Mölndal, Sweden). Abbreviation expansions appear in caption to Figure 1. VaD indicates vascular dementia.

Figure 1A and B show mean [^18^F]RO948 SUVR images across diagnostic groups. Voxelwise comparisons are shown in Figure 1C and eFigure 3 in the Supplement. Familywise error–corrected results are shown in eFigure 4 in the Supplement. Compared with Aβ-negative cognitively unimpaired controls, [^18^F]RO948 retention was higher in patients with AD dementia (widespread), Aβ-positive MCI (temporal/parietal cortices), dementia with Lewy bodies (temporal/lateral parietal cortices), svPPA (temporal pole), and progressive supranuclear palsy (globus pallidus) (eFigure 3 in the Supplement). Figure 2A-D show [^18^F]RO948 SUVRs across all diagnostic groups for the 4 different tau-imaging ROIs (I-II, III-IV, I-IV, and V-VI). Irrespective of clinical diagnosis, tau PET positivity was almost exclusively observed in Aβ-positive individuals. Exceptions included *MAPT* R406W mutation carriers: one individual without cognitive impairment and one patient with vascular dementia with isolated tau PET positivity in the I-II ROI (entorhinal cortex). In contrast with the findings of previous studies with [^18^F]flortaucipir,^11,39,40,41^ no patient with svPPA was tau PET positive across the cortical ROIs (Figure 2). Results were essentially the same when using partial volume error–corrected [^18^F]RO948 SUVR data (eFigure 5 and eFigure 6 in the Supplement). The [^18^F]RO948 SUVRs using more liberal cutoffs and by age are shown in eFigure 7 and eFigure 8 in the Supplement, and [^18^F]RO948 SUVRs in primary sensory and motor cortices are shown in eFigure 9 in the Supplement.

### AD Dementia vs Non-AD Disorders

The [^18^F]RO948 SUVRs could distinguish AD dementia from non-AD disorders (Table 2). Group separation was best using the I-IV ROI, with an AUC of 0.97 (95% CI, 0.95-0.99), 91.3% (95% CI, 87.2%-94.9%) correctly classified, sensitivity of 91.9% (95% CI, 85.9%-97.0%), and specificity of 90.6% (95% CI, 84.4%-95.8%). Comparable findings were observed using the I-II and III-IV ROIs. While the V-VI ROI showed a similarly high AUC (0.92; 95% CI, 0.88-0.96), the percentage of correctly classified participants was lower (78.5%; 95% CI, 73.3%-83.6%) owing to a clearly lower sensitivity of 59.6% (95% CI, 50.5%-69.7%) but with only somewhat better specificity (97.9%; 95% CI, 94.8%-100%). Positivity in the V-VI ROI had a higher positive likelihood ratio (19.07; 95% CI, 6.19-58.77) compared with the other ROIs (range, 6.79-8.73). The diagnostic performance of [^18^F]RO948 when separating AD from different types of non-AD disorders was high except when distinguishing AD from dementia with Lewy bodies, where specificity was low (70.0%; 95% CI, 52.3%-87.0%) (eTable 6 in the Supplement). Considering that mixed dementia with Lewy bodies and AD are common,^42^ results obtained when the dementia with Lewy bodies group is subdivided by Aβ status are shown in eFigure 10 and eFigure 11 in the Supplement. Furthermore, the diagnostic performance of [^18^F]RO948 using SUVRs in individual tau-imaging ROIs is summarized in eTable 7 in the Supplement. Diagnostic performance for tau-imaging ROIs using lower cutoffs for [^18^F]RO948 SUVRs (mean [SD] +1.5 [2] in Aβ-negative young controls) are summarized in eTable 8 and eFigure 7 in the Supplement.

**Table 2.** Diagnostic Performance of RO948 F 18 PET in Distinguishing AD Dementia and Aβ-Positive Mild Cognitive Impairment From Non-AD Neurodegenerative Disorders

Tau-imaging ROI	Cutoff^a^	Performance (95% CI)					
		AUC	Agreement	Sensitivity	Specificity	Likelihood ratio	
						Positive	Negative
**AD dementia (n = 100) vs cognitively unimpaired controls (n = 257)**^**b**^							
Braak stage I-II	>1.48	0.97 (0.95-0.98)	92.3 (89.2-94.8)	91.9 (85.9-97.0)	92.4 (88.9-95.4)	11.31 (7.40-17.28)	0.09 (0.05-0.17)
Braak stage III-IV	>1.36	0.97 (0.95-0.99)	92.5 (89.9-95.3)	85.9 (78.8-91.9)	95.1 (92.4-97.7)	15.26 (9.13-25.53)	0.14 (0.08-0.23)
Braak stage I-IV	>1.36	0.98 (0.96-0.99)	93.9 (91.4-96.4)	91.0 (84.9-96.0)	95.1 (92.4-97.3)	17.20 (10.10-29.31)	0.10 (0.05-0.18)
Braak stage V-VI	>1.35	0.91 (0.87-0.95)	87.9 (85.1-90.6)	59.6 (49.9-69.7)	98.5 (97.0-99.6)	48.87 (15.69-152.24)	0.41 (0.32-0.52)
**AD dementia (n = 100) vs non-AD disorders (n = 102)**^**b**^^,^^**c**^							
Braak stage I-II	>1.48	0.96 (0.93-0.99)	90.3 (86.1-94.4)	92.9 (87.9-97.0)	87.5 (80.0-93.8)	6.79 (4.08-11.29)	0.09 (0.05-0.18)
Braak stage III-IV	>1.36	0.96 (0.94-0.98)	88.7 (84.2-92.9)	86.9 (79.8-92.9)	90.6 (84.4-95.8)	8.34 (4.62-15.07)	0.15 (0.09-0.24)
Braak stage I-IV	>1.36	0.97 (0.95-0.99)	91.3 (87.2-94.9)	91.9 (85.9-97.0)	90.6 (84.4-95.8)	8.73 (4.84-15.74)	0.10 (0.05-0.19)
Braak stage V-VI	>1.35	0.92 (0.88-0.96)	78.5 (73.3-83.6)	59.6 (50.5-69.7)	97.9 (94.8-100)	19.07 (6.19-58.77)	0.42 (0.33-0.53)
**Aβ-positive MCI (n = 96) vs cognitively unimpaired controls (n = 257)**^**b**^							
Braak stage I-II	>1.48	0.78 (0.72-0.84)	80.6 (77.3-83.9)	46.9 (37.5-56.3)	92.6 (89.6-95.6)	5.77 (3.60-9.23)	0.58 (0.48-0.70)
Braak stage III-IV	>1.36	0.77 (0.72-0.83)	79.8 (76.5-82.8)	36.5 (27.1-45.8)	95.2 (92.6-97.4)	6.41 (3.61-11.36)	0.67 (0.58-0.79)
Braak stage I-IV	>1.36	0.80 (0.75-0.85)	80.1 (76.8-83.1)	37.5 (28.1-47.0)	95.2 (92.6-97.8)	12.22 (7.06-21.17)	0.37 (0.28-0.49)
Braak stage V-VI	>1.35	0.59 (0.52-0.66)	76.2 (74.3-78.1)	13.0 (6.3-18.8)	99.0 (97.0-100)	11.06 (3.22-37.95)	0.88 (0.81-0.95)
**Aβ-positive MCI (n = 96) vs non-AD disorders (n = 102)**^**b**^^,^^**c**^							
Braak stage I-II	>1.48	0.72 (0.65-0.79)	68.0 (61.4-73.6)	47.0 (37.1-57.3)	87.1 (80.2-94.1)	3.46 (2.00-5.99)	0.61 (0.50-0.75)
Braak stage III-IV	>1.36	0.71 (0.64-0.78)	64.0 (58.4-70.0)	36.5 (27.1-45.8)	90.1 (84.2-95.1)	3.50 (1.84-6.66)	0.71 (0.60-0.84)
Braak stage I-IV	>1.36	0.73 (0.66-0.80)	65.0 (59.0-70.1)	37.5 (28.1-46.9)	90.1 (84.2-95.1)	6.20 (3.39-11.35)	0.40 (0.30-0.52)
Braak stage V-VI	>1.35	0.59 (0.53-0.69)	56.0 (52.3-60.0)	13.0 (6.3-19.8)	97.0 (94.1-100)	4.33 (1.28-14.72)	0.89 (0.82-0.97)

Abbreviations: Aβ, amyloid β; AD, Alzheimer disease; AUC, area under the curve; MCI, mild cognitive impairment; PET, positron emission tomographic; ROI, region of interest; SUVR, standardized uptake value ratio.

^a^ Thresholds for tau PET positivity within composite ROIs were established using the mean SUVR within a given ROI plus 2.5 SDs among 17 Aβ-negative young (age, 20-40) control individuals.

^b^ Braak stage I-II, entorhinal cortex; III-IV, inferior/middle temporal, fusiform gyrus, parahippocampal cortex, and amygdala; I-IV; and V-VI, widespread neocortical.

^c^ Neurodegenerative disorders other than AD.

### Aβ-Positive MCI vs Non-AD Disorders

Compared to the AD dementia group, the discriminative ability of the [^18^F]RO948 SUVR across ROIs was, as expected, lower for the separation of Aβ-positive MCI from the non-AD group (Table 2). Using the I-IV ROI, which provided the best separation for both contrasts, the AUC for Aβ-positive MCI vs the non-AD group was 0.73 (95% CI, 0.66-0.80), the percentage of correctly classified participants was 65.0% (95% CI, 59.0%-70.1%), sensitivity was 37.5% (95% CI, 28.1%-46.9%), and specificity was 90.1% (95% CI, 84.2%-95.1%).

### [^18^F]RO948 vs MRI and CSF Markers

**Figure 3.**
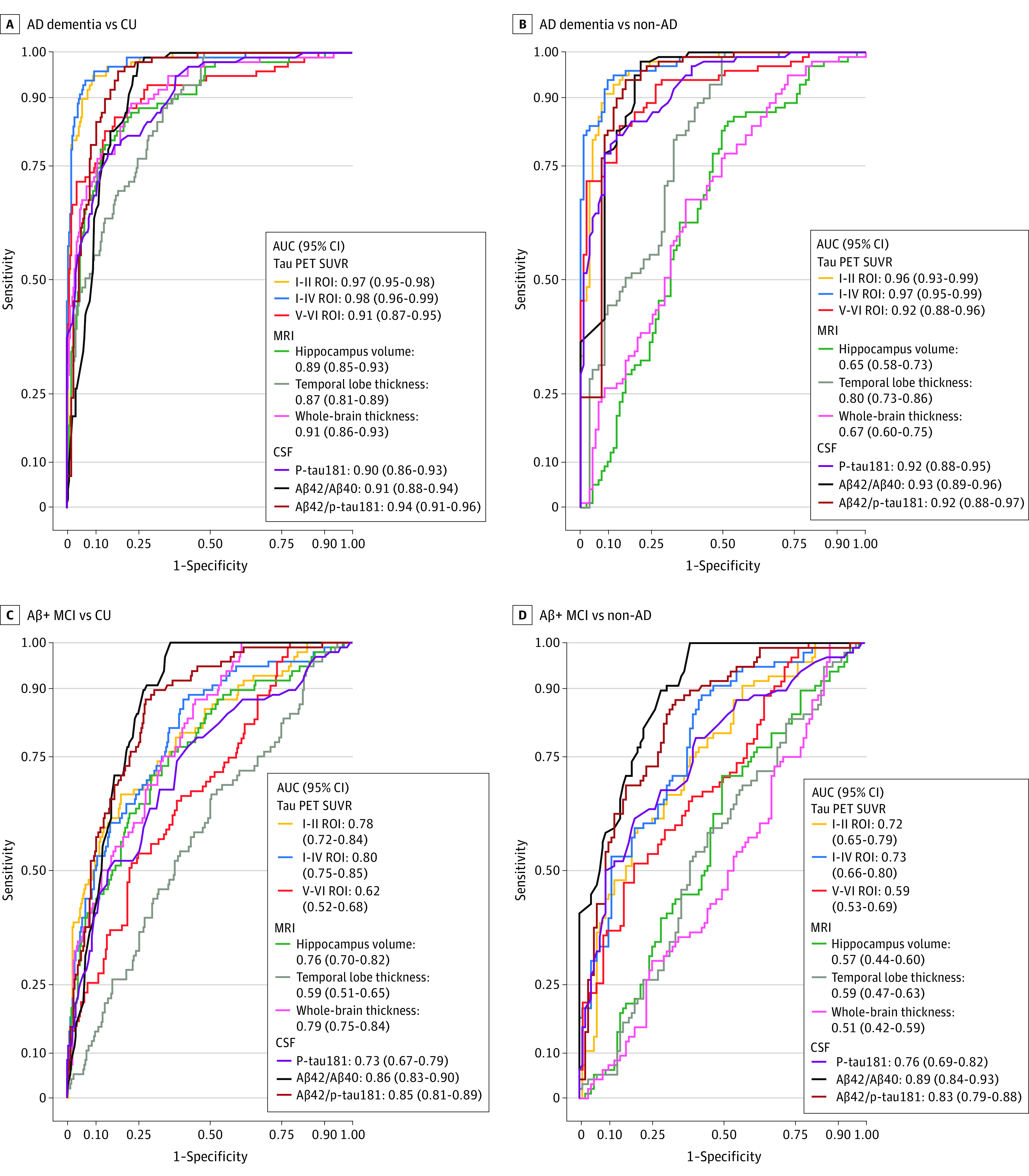
Plots From Receiver Operating Characteristic Analyses for RO948 F 18 ([^18^F]RO948) Tau Positron Emission Tomographic (PET) Tracers, Magnetic Resonance Imaging (MRI), and Cerebrospinal Fluid (CSF) Measures for Distinguishing Alzheimer Disease (AD) Dementia and Amyloid β (Aβ)-Positive Mild Cognitive Impairment (MCI) From Cognitively Unimpaired Controls and Non-AD Neurodegenerative Disorders Receiver operating characteristic curves are shown for the following groups: AD dementia vs cognitively unimpaired (CU) controls (A) and non-AD disorders (B), and Aβ-positive MCI vs CU controls (C) and non-AD disorders (D). The III-IV region of interest, omitted owing to its similar performance to I-IV, had the following area under the curve (AUC) values: AD dementia vs cognitively unimpaired controls, AUC = 0.97 (95% CI, 0.95-0.99); AD dementia vs non-AD: AUC = 0.96 (95% CI, 0.94-0.98); Aβ-positive MCI vs cognitively unimpaired controls: AUC = 0.77 (95% CI, 0.72-0.83); and Aβ-positive MCI vs non-AD: AUC = 0.71 (95% CI, 0.65-0.78). ROI indicates region of interest; SUVR, standardized uptake value ratio.

Figure 3A shows that the highest AUC was obtained using the I-IV ROI (0.98; 95% CI, 0.96-0.99), which outperformed MRI (highest AUC = 0.91 (95% CI, 0.86-0.93) using whole-brain cortical thickness) and CSF measures (highest AUC = 0.94, 95% CI, 0.91-0.96) using Aβ42/p-tau181. Figure 3B shows that AUCs for [^18^F]RO948 (I-II ROI: 0.96; 95% CI, 0.93-0.99; I-IV ROI: 0.97; 95% CI, 0.95-0.99; and V-VI ROI: 0.92; 95% CI, 0.88-0.96) were significantly higher than those for MRI (AUC hippocampal volume: 0.65; 95% CI, 0.58-0.73; temporal lobe thickness: 0.80; 95% CI, 0.73-0.86; and whole-brain thickness: 0.67; 95% CI, 0.60-0.75; *P* < .001 for all comparisons). The AUCs were also significantly higher than the CSF AD biomarkers (p-tau181: 0.92; 95% CI, 0.88-0.95; *P* < .05; Aβ42/Aβ40: 0.93; 95% CI, 0.89-0.96; *P* < .05; and Aβ42/p-tau181: 0.92; 95% CI, 0.88-0.97; *P* < .05 compared with all [^18^F]RO948 ROIs) for distinguishing AD dementia from non-AD. When comparing patients with AD dementia with both controls with no cognitive impairment and patients with non-AD disorders, [^18^F]RO948 SUVR measures also carried higher specificity (Table 2; eTable 9 in the Supplement). In participants with AD dementia vs controls with no cognitive impairment, the [^18^F]RO948 SUVR within the I-IV ROI had a specificity of 95.1% (95% CI, 92.4%-97.3%) compared with 63.9% (95% CI, 58.2%-69.6%) for CSF. With Aβ42/Aβ40, similar findings were seen for AD dementia vs non-AD with a specificity of the [^18^F]RO948 SUVR within the I-IV ROI of 90.6% (95% CI, 84.4%-95.8%) compared with 61.5% (95% CI, 52.1%-70.8%) for Aβ42/Aβ40. Similar findings also were obtained when looking at Aβ-positive MCI. For the contrast Aβ-positive MCI vs non-AD disorders, AUCs for [^18^F]RO948 were higher than for MRI (range, [^18^F]RO948 ROIs, 0.59-0.73; range, MRI, 0.51-0.59; *P* < .001 for all comparisons), but CSF ratio measures provided the best group separation (Aβ42/Aβ40, 0.89 [95% CI, 0.84-0.93]; Aβ42/p-tau181, 0.83 [95% CI, 0.79-0.88]). Cerebrospinal fluid p-tau181 by diagnostic group and its association with the [^18^F]RO948 SUVR in tau-imaging ROIs are shown in eFigure 12 and eFigure 13 in the Supplement. The III-IV region, omitted owing to its similar performance to I-IV, had the following AUC values: AD dementia vs cognitively unimpaired controls, AUC = 0.97 (95% CI, 0.95-0.99); AD dementia vs non-AD, AUC = 0.96 (95% CI, 0.94-0.98); Aβ-positive MCI vs cognitively unimpaired controls, AUC = 0.77 (95% CI, 0.72-0.83); and Aβ-positive MCI vs non-AD, AUC = 0.71 (95% CI, 0.64-0.78).

### [^18^F]RO948 and [^18^F]Flortaucipir SUVRs in svPPA

All 7 patients with svPPA exhibited low retention of [^18^F]RO948 (Figure 2). Visual inspection of [^18^F]RO948 and [^18^F]flortaucipir SUVR images in the 3 patients with svPPA who underwent PET studies with both tracers suggested higher retention of [^18^F]flortaucipir (eFigure 14A in the Supplement). This finding was confirmed by a voxelwise subtraction analysis that showed [^18^F]flortaucipir SUVRs to be higher than those for [^18^F]RO948 ([^18^F]flortaucipir − [^18^F]RO948), particularly within the anterior temporal lobe bilaterally (eFigure 14B in the Supplement). The reverse operation ([^18^F]R0948 − ([^18^F]flortaucipir) showed higher SUVR values for [^18^F]R0948 only within the retina and skull/meninges (eFigure 14C in the Supplement). Higher retention of [^18^F]flortaucipir was further supported by comparison of SUVR values within the anterior temporal lobe ROI (eTable 10 in the Supplement).

### Retention of [^18^F]RO948 in the Skull/Meninges

Elevated retention of [^18^F]R0948 in the skull/meninges (confluent signal on visual inspection, with SUVR>2.5)^24^ was seen in 4.4% of BioFINDER-2 participants (eTable 11 in the Supplement). Furthermore, to assess the association between this off-target signal and diagnostic performance, receiver operating characteristic analyses were repeated, excluding participants with high SUVRs in the skull/meninges. Resulting AUC values did not differ significantly from those including all participants (eTable 11 in the Supplement).

## Discussion

Overall findings with [^18^F]RO948 supported in vitro findings of high specificity of [^18^F]RO948 for AD-type neurofibrillary tangles.^18,20^ Within the I-II (entorhinal cortex) ROI, primary age-related tauopathy^43^ may account for the participants (1 with no cognitive impairment and 1 with vascular dementia) who showed tau PET positivity using [^18^F]RO948 (hypothetically, since primary age-related tauopathy cannot be diagnosed during life). Across limbic (III-IV and I-IV) and neocortical (V-VI) ROIs, tau positivity was seen in Aβ-positive cases and in 2 carriers of an *MAPT* R406W mutation associated with the formation of AD-like PHFs.^44^ In general, [^18^F]RO948 did not bind significantly in the included non-AD disorders. Some cortical signal was seen in dementia with Lewy bodies, but this signal appeared to be associated with Aβ-positive cases. Alzheimer disease is a frequent concomitant pathology in dementia with Lewy bodies,^42,45^ possibly owing to the cross-seeding of α-synuclein, Aβ, and tau pathologies.^46,47^

Imaging with Aβ PET and CSF ratios including Aβ42 shows high sensitivity to Aβ pathology, changing approximately 15 to 25 years before the onset of cognitive decline.^48,49^ Owing to the high prevalence of Aβ positivity in cognitively unimpaired, older, healthy individuals and in older persons with non-AD neurodegenerative diseases, Aβ biomarkers are generally used to rule out—as opposed to rule in—AD as the cause of cognitive impairment.^50^ Indeed, Aβ positivity has been shown to increase with age in most non-AD disorders, in which Aβ may be a secondary condition.^45^ In a recent study,^9^ [^18^F]flortaucipir showed greater discriminative accuracy in older populations compared with Aβ biomarkers. Our finding of higher specificity for [^18^F]RO948 than for CSF Aβ42/Aβ40, when distinguishing AD dementia from no cognitive impairment and non-AD diseases, is consistent with this study and earlier findings that tau PET positivity is associated with the onset of cognitive decline and dementia.^51^ Together, these findings appear to support the notion that tau PET is a valuable addition to Aβ biomarkers in the workup of early-onset dementia and as a stand-alone test in patients with late-onset dementia where Aβ pathology is frequent. A single tau PET scan may preclude the need for Aβ PET or CSF AD biomarkers, as well as fluorodeoxyglucose F 18 PET, in the diagnostic workup of amnestic dementia (eFigure 15 in the Supplement) given the finding that tau PET positivity was virtually confined to Aβ-positive cases and studies showing a high overlap between tau PET and hypometabolism.^52,53^

The diagnostic performance of [^18^F]RO948 was lower when differentiating Aβ-positive MCI from other conditions compared with AD dementia (Figure 1). Tau PET may thus have greater value for differential diagnosis of dementia as opposed to early disease where CSF biomarkers may have better sensitivity.^54^ Longitudinal studies are needed to address which biomarker (tau PET, Aβ PET, or CSF AD biomarkers) is best for estimation of progression to AD dementia in MCI.

Compared with [^18^F]RO948, MRI and CSF measures performed less well in distinguishing AD dementia from non-AD disorders. The CSF AD biomarkers, including CSF Aβ42, and the ratios including Aβ42, may plateau early in the course of the disease and best act as disease state markers, reflecting the intensity of the disease process.^54^ Tau PET and MRI-based atrophy measures, by contrast, change in a somewhat continuous fashion throughout the disease, reflecting disease progression.^54^ Tau accumulation and cortical atrophy are offset processes, however, with PHF tau thought to accumulate upstream from neurodegeneration.^55,56^ As such, the accumulation of tau aggregates may be the most dynamic of the 3 processes within the present cohort, with CSF markers already fully changed in participants without cognitive impairment but with Aβ pathology. While our findings are consistent with earlier work showing that tau PET outperformed MRI in differentiating AD from non-AD disorders,^9^ the present study is, to our knowledge, the first to report findings from a large number of individuals using a second-generation tau tracer and directly compare the diagnostic performance of tau PET, MRI, and CSF markers for AD vs other neurodegenerative disorders. The optimal diagnostic marker may depend on disease phase. Tau PET may prove best for the differentiation of early AD dementia from other neurodegenerative disorders, and CSF and Aβ PET may better aid differential diagnosis in preclinical and prodromal AD.

On the basis of postmortem studies, TDP-43 type C pathology is the primary substrate of svPPA in most patients, although AD and Pick disease have also been shown as causes.^12,57,58,59^ Several in vivo PET studies^9,11,39,40^ have identified increased retention of [^18^F]flortaucipir in svPPA despite autoradiographic studies showing minimal to no binding of [^18^F]flortaucipir to TDP-43 pathology.^60,61,62^ Off-target binding to a neurodegenerative process other than tau has been proposed as an explanation,^11^ as has binding to monoamine oxidase B expressed by reactive astrocytes,^63^ although in vivo findings are mixed with respect to this hypothesis.^39,40,64,65^ Although the lower signal seen with [^18^F]RO948 suggests a more favorable specificity profile compared with [^18^F]flortaucipir, this observation can only be considered preliminary owing to the lack of neuropathologic confirmation of underlying TDP-43 pathology in the included svPPA cases and the fact that an [^18^F]RO948 signal was seen, albeit at a low level. Although [^18^F]RO948 has been shown to have negligible retention in vivo in the basal ganglia, thalamus, and choroid plexus,^21^ retention in the skull/meninges—a feature seen in the subtraction analysis—has been reported^21,24^ and has also been shown to affect other tau tracers, such as MK6240 F 18.^15,16^ Although the cause of this off-target binding is unclear, the frequency of elevated [^18^F]RO948 SUVRs in the skull/meninges was relatively low and has been shown to be stable over time.^24^ In the present study, the diagnostic performance of [^18^F]RO948 did not improve after participants with high off-target binding were removed.

### Strengths and Limitations

Strengths of this study include the large sample size, the use of a novel tau tracer with image acquisition performed in a single PET center, and the multiple imaging and fluid biomarkers used for comparisons of diagnostic performance. This study has limitations. One limitation was that clinical diagnosis was considered to be the reference standard, as autopsy data were not available. Because the clinical diagnosis of AD may be inaccurate,^66^ the standard used for comparison in the receiver operating characteristic analysis was suboptimal. Furthermore, the number of participants in several of the non-AD subgroups was small (eg, multiple system atrophy and vascular dementia). Findings in these groups should be considered preliminary. Cerebrospinal fluid Aβ42/Aβ40 vs Aβ PET was used to define Aβ status. This difference is unlikely to have biased group assignment (Aβ-positive/Aβ-negative) considering the high concordance with Aβ PET.^67^ Consensus is lacking regarding the optimal approach to determine cutoffs for tau PET SUVRs. Our approach was conservative and consistent with those used in comparable studies.^35^ The generalizability of our findings to additional second-generation tau tracers remains uncertain until similar studies are performed using these compounds.

## Conclusions

The findings suggest that [^18^F]RO948 PET was able to discriminate AD dementia from other neurodegenerative diseases in a memory clinic setting and was superior to CSF p-tau181, Aβ42/Aβ40, Aβ42/p-tau181, and MRI measures. The results suggest that [^18^F]RO948 has high specificity for AD-type PHF tau pathology outside the medial temporal lobe that is seen only in the context of Aβ positivity and certain *MAPT* mutations.

## Notes

Supplement. eMethods 1. Inclusion and Exclusion Criteria for the Swedish BioFINDER 2 Study eMethods 2. Additional Details About [18F]RO948 Preprocessing eMethods 3. Temporal Regions of Interest in Semantic Variant Primary Progressive Aphasia eMethods 4. Additional Details About CSF Procedures eTable 1. Demographics of Young (Age 20-40) Aβ-Negative Controls Used to Set [18F]RO948 SUVR Cutoffs eResults. Participant Characteristics eTable 2. Demographics for Non-Alzheimer Disease Subgroups eTable 3. Partial Volume Error Corrected [18F]RO948 SUVR Data for Cognitively Unimpaired Controls and Mild Cognitive Impairment, Alzheimer Disease Dementia and Non-Alzheimer Disease Disorder Patients eTable 4. Partial Volume Error Corrected [18F]RO948 SUVR Data for Non-AD Subgroups eTable 5. Demographics for svPPA Cases eTable 6. Diagnostic Performance of [18F]RO948 SUVR Using the Tau-Imaging I-IV ROI for AD Dementia and Aβ-Positive MCI vs Non-AD Disorders eTable 7. Diagnostic Performance of [18F]RO948 SUVR Using Individual Tau Imaging ROIs eTable 8. AUC Values for [18F]RO948 SUVR Using Different Cutoffs eTable 9. Diagnostic Performance of CSF Aβ42/Aβ40 for AD Dementia and Aβ Positive MCI vs Other Non-AD Disorders and CU Controls eTable 10. [18F]Flortaucipir and [18F]RO948 SUVR Values in Temporal and Primary Somatosensory Cortex ROIs for Semantic Variant Primary Progressive Aphasia eTable 11. Area Under the Receiver Operating Characteristic Curve Values for [18F]RO948 SUVR in Tau Imaging ROIs With and Without Subjects Showing High Skull/Meningeal Signal eFigure 1. Tau PET Imaging Composite ROIs Approximating the Braak Post-Mortem Staging Scheme for Tau Pathology eFigure 2. Mean [18F]RO948 Images and Scatterplots for the Young (Age 20-40) Aβ-Negative Controls Used to Set Cutoffs for [18F]RO948 SUVR Across Tau-Imaging ROIs eFigure 3. Voxelwise Group Differences in [18F]RO948 SUVR eFigure 4. Voxelwise Group Differences in [18F]RO948 SUVR Using Family Wise Error Corrected Data eFigure 5. Partial Volume Corrected [18F]RO948 Standardized Uptake Values Ratios (SUVRs) Across Diagnostic Groups Within Tau-Imaging ROIs eFigure 6. Concordance Plots Between Partial Volume Corrected [18F]RO948 Standardized Uptake Values Ratios (SUVRs) and CSF Aβ42/Aβ40 eFigure 7. [18F]RO948 SUVR Across Tau-Imaging ROIs Using Lower Cutoffs eFigure 8. [18F]RO948 SUVR Across Tau-Imaging ROIs by Age (Above and Below 65) eFigure 9. [18F]RO948 SUVR in Primary Somatosensory and Motor Cortices eFigure 10. Mean [18F]RO948 Standardized Uptake Values Ratios (SUVR) Across Diagnostic Groups (DLB Subdivided by Aβ-Status) Within Tau-Imaging ROIs eFigure 11. Plots From Receiver Operating Characteristic Analyses ([18F]RO948, MRI- and CSF-Measures) for Distinguishing AD Dementia and Aβ-Positive MCI Fm Non-AD Neurodegenerative Disorders eFigure 12. CSF P-Tau181 Levels by Diagnostic Group eFigure 13. CSF P-Tau181 Levels Across Tau-Imaging ROIs eFigure 14. [18F]RO948 and [18F]Flortaucipir PET in Semantic Variant Primary Progressive Aphasia eFigure 15. Decision Tree Outlining the Potential Clinical Utility of Tau-PET Imaging Across Different Dementia Disorders, Including Alzheimer Disease
