# Supplementary material for: Diagnostic Performance of RO948 F 18 Tau Positron Emission Tomography in the Differentiation of Alzheimer Disease From Other Neurodegenerative Disorders
Source: JAMA Neurol. 2020 May 11;77(8):1–12. doi: 10.1001/jamaneurol.2020.0989 (PMC7215644; doi:10.1001/jamaneurol.2020.0989)
Supplement: Supplement. — eMethods 1. Inclusion and Exclusion Criteria for the Swedish BioFINDER 2 Study eMethods 2. Additional Details About [18F]RO948 Preprocessing eMethods 3. Temporal Regions of Interest in Semantic Variant Primary Progressive Aphasia eMethods 4. Additional Details About CSF Procedures eTable 1. Demographics of Young (Age 20-40) Aβ-Negative Controls Used to Set [18F]RO948 SUVR Cutoffs eResults. Participant Characteristics eTable 2. Demographics for Non-Alzheimer Disease Subgroups eTable 3. Partial Volume Error Corrected [18F]RO948 SUVR Data for Cognitively Unimpaired Controls and Mild Cognitive Impairment, Alzheimer Disease Dementia and Non-Alzheimer Disease Disorder Patients eTable 4. Partial Volume Error Corrected [18F]RO948 SUVR Data for Non-AD Subgroups eTable 5. Demographics for svPPA Cases eTable 6. Diagnostic Performance of [18F]RO948 SUVR Using the Tau-Imaging I-IV ROI for AD Dementia and Aβ-Positive MCI vs Non-AD Disorders eTable 7. Diagnostic Performance of [18F]RO948 SUVR Using Individual Tau Imaging ROIs eTable 8. AUC Values for [18F]RO948 SUVR Using Different Cutoffs eTable 9. Diagnostic Performance of CSF Aβ42/Aβ40 for AD Dementia and Aβ Positive MCI vs Other Non-AD Disorders and CU Controls eTable 10. [18F]Flortaucipir and [18F]RO948 SUVR Values in Temporal and Primary Somatosensory Cortex ROIs for Semantic Variant Primary Progressive Aphasia eTable 11. Area Under the Receiver Operating Characteristic Curve Values for [18F]RO948 SUVR in Tau Imaging ROIs With and Without Subjects Showing High Skull/Meningeal Signal eFigure 1. Tau PET Imaging Composite ROIs Approximating the Braak Post-Mortem Staging Scheme for Tau Pathology eFigure 2. Mean [18F]RO948 Images and Scatterplots for the Young (Age 20-40) Aβ-Negative Controls Used to Set Cutoffs for [18F]RO948 SUVR Across Tau-Imaging ROIs eFigure 3. Voxelwise Group Differences in [18F]RO948 SUVR eFigure 4. Voxelwise Group Differences in [18F]RO948 SUVR Using Family Wise Error Corrected Data eFigure 5. Partial Vol [file jamaneurol-77-955-s001.pdf]

# Supplementary Online Content

Leuzy A, Smith R, Ossenkoppele R, et al. Diagnostic performance of RO948 F 18 tau positron emission tomography in the differentiation of Alzheimer disease from other neurodegenerative disorders. *JAMA Neurol*. Published online May 11, 2020.
doi:10.1001/jamaneurol.2020.0989

**eMethods 1.** Inclusion and Exclusion Criteria for the Swedish BioFINDER 2 Study

**eMethods 2.** Additional Details About [$^{18}$F]RO948 Preprocessing

**eMethods 3.** Temporal Regions of Interest in Semantic Variant Primary Progressive Aphasia

**eMethods 4.** Additional Details About CSF Procedures

**eTable 1.** Demographics of Young (Age 20-40) Aβ-Negative Controls Used to Set [$^{18}$F]RO948 SUVR Cutoffs

**eResults.** Participant Characteristics

**eTable 2.** Demographics for Non-Alzheimer Disease Subgroups

**eTable 3.** Partial Volume Error Corrected [$^{18}$F]RO948 SUVR Data for Cognitively Unimpaired Controls and Mild Cognitive Impairment, Alzheimer Disease Dementia and Non-Alzheimer Disease Disorder Patients

**eTable 4.** Partial Volume Error Corrected [$^{18}$F]RO948 SUVR Data for Non-AD Subgroups

**eTable 5.** Demographics for svPPA Cases

**eTable 6.** Diagnostic Performance of [$^{18}$F]RO948 SUVR Using the Tau-Imaging I-IV ROI for AD Dementia and Aβ-Positive MCI versus Non-AD Disorders

**eTable 7.** Diagnostic Performance of [$^{18}$F]RO948 SUVR Using Individual Tau Imaging ROIs

**eTable 8.** AUC Values for [$^{18}$F]RO948 SUVR Using Different Cutoffs

**eTable 9.** Diagnostic Performance of CSF $A\beta_{42}/A\beta_{40}$ for AD Dementia and Aβ Positive MCI versus Other Non-AD Disorders and CU Controls

**eTable 10.** [$^{18}$F]Flortaucipir and [$^{18}$F]RO948 SUVR Values in Temporal and Primary Somatosensory Cortex ROIs for Semantic Variant Primary Progressive Aphasia

**eTable 11.** Area Under the Receiver Operating Characteristic Curve Values for [$^{18}$F]RO948 SUVR in Tau Imaging ROIs With and Without Subjects Showing High Skull/Meningeal Signal

**eFigure 1.** Tau PET Imaging Composite ROIs Approximating the Braak Post-Mortem Staging Scheme for Tau Pathology

**eFigure 2.** Mean [$^{18}$F]RO948 Images and Scatterplots for the Young (Age 20-40) Aβ-Negative Controls Used to Set Cutoffs for [$^{18}$F]RO948 SUVR Across Tau-Imaging ROIs

**eFigure 3.** Voxelwise Group Differences in [$^{18}$F]RO948 SUVR

**eFigure 4.** Voxelwise Group Differences in [$^{18}$F]RO948 SUVR Using Family Wise Error Corrected Data

**eFigure 5.** Partial Volume Corrected [$^{18}$F]RO948 Standardized Uptake Values Ratios (SUVRs) Across Diagnostic Groups Within Tau-Imaging ROIs

**eFigure 6.** Concordance Plots Between Partial Volume Corrected [$^{18}$F]RO948 Standardized Uptake Values Ratios (SUVRs) and CSF $A\beta_{42}/A\beta_{40}$

**eFigure 7.** [$^{18}$F]RO948 SUVR Across Tau-Imaging ROIs Using Lower Cutoffs

**eFigure 8.** [$^{18}$F]RO948 SUVR Across Tau-Imaging ROIs by Age (Above and Below 65)

**eFigure 9.** [$^{18}$F]RO948 SUVR in Primary Somatosensory and Motor Cortices

**eFigure 10.** Mean [$^{18}$F]RO948 Standardized Uptake Values Ratios (SUVR) Across Diagnostic Groups (DLB Subdivided by Aβ-Status) Within Tau-Imaging ROIs

**eFigure 11.** Plots From Receiver Operating Characteristic Analyses ([$^{18}$F]RO948, MRI- and CSF-Measures) for Distinguishing AD Dementia and Aβ-Positive MCI Fm Non-AD Neurodegenerative Disorders

**eFigure 12.** CSF $\text{P-Tau}_{181}$ Levels by Diagnostic Group

**eFigure 13.** CSF $\text{P-Tau}_{181}$ Levels Across Tau-Imaging ROIs

**eFigure 14.** [$^{18}$F]RO948 and [$^{18}$F]Flortaucipir PET in Semantic Variant Primary Progressive Aphasia

**eFigure 15.** Decision Tree Outlining the Potential Clinical Utility of Tau-PET Imaging Across Different Dementia Disorders, Including Alzheimer Disease

This supplementary material has been provided by the authors to give readers additional information about their work.

**eMethods 1.** Inclusion and exclusion criteria for the Swedish BioFINDER 2 study

**Diagnostic criteria**

The BioFINDER-2 study enrolls participants in five sub-cohorts; Cohort A and B includes neurologically and cognitively healthy controls. The inclusion criteria are: i) ages 40-65 years (cohort A) and ages 66-100 years (cohort B); ii) absence of cognitive symptoms as assessed by a physician with special interest in cognitive disorders; iii) MMSE score 27-30 (A) or 26-30 (cohort B) at screening visit; iv) do not fulfill the criteria for MCI or any dementia according to DSM-5 (American Psychiatric Association, 2013); v) fluent in Swedish. The recruitment process of cohorts A and B is designed to build two study populations with 50% *APOE* ε4 carriers in each.

Cohort C comprises participants with subjective cognitive deficits (SCD) or minor neurocognitive impairment (MCI) (the latter according to DSM-5 (American Psychiatric Association,2013). Inclusion criteria are: i) Age 40-100 years; ii) referred to the memory clinics due to cognitive symptoms; iii) MMSE score of 24 – 30 points; iv) does not fulfill the criteria for any dementia (major neurocognitive disorder) according to DSM-5 (American Psychiatric Association, 2013), v) fluent in Swedish. In accordance with the research framework by the National Institute on Aging-Alzheimer's Association (Jack *et al.*, 2018) study participants with SCD were analyzed together with the cognitively healthy participants (and combined in the cognitively unimpaired group). Participants were classified as having MCI if they performed worse than -1.5 SD in any cognitive domain according to age and education stratified test norms. The neuropsychological battery covered the domains attention/executive function (Trail Making Test A and B, Symbol Digit Modalities Test, and AQT), memory (10 word immediate and delayed recall from the Alzheimer's Disease Assessment Scale [ADAS]), verbal ability (verbal fluency and the short version of the Boston Naming Test) and visuospatial function (incomplete letters and cube analysis from the Visual Object and Space Perception battery). Those that were not classified as MCI were considered to have SCD.

Cohort D consists of participants with dementia due to AD. Inclusion criteria are: i) Age 40-100 years; ii) referred to the memory clinics due to cognitive symptoms; iii) MMSE score of ≥12 points; iv) fulfill the DSM-5 criteria for dementia (major neurocognitive disorder) due to Alzheimer's disease (American Psychiatric Association, 2013); v) fluent in Swedish.

Cohort E covers other non-AD dementias and neurodegenerative disorders. Inclusion criteria are: i) Age 40-100 years; ii) fulfillment of criteria for dementia (major neurocognitive disorder) due to frontotemporal dementia (American Psychiatric Association, 2013), Parkinson's disease with dementia, dementia with Lewy Bodies or vascular dementia (American Psychiatric Association, 2013) alternatively the criteria for Parkinson's disease (Gelb *et al.*, 1999), progressive supranuclear palsy (Hoglinger *et al.*, 2017), multiple system atrophy (Gilman *et al.*, 2008), or semantic variant primary progressive aphasia (Gorno-Tempini *et al.*, 2011); iii) fluent in Swedish.
Exclusion criteria for all sub-cohorts are: i) significant unstable systemic illness that makes it difficult to participate in the study; ii) current significant alcohol or substance misuse; iii) refusing lumbar puncture, MRI or PET.

**eReferences**


American Psychiatric Association. Diagnostic and Statistical Manual of Mental Disorders, Fifth Edition (DSM-5). Arlington, VA; 2013.
Gelb DJ, Oliver E, Gilman S. Diagnostic criteria for Parkinson disease. Arch Neurol 1999; 56(1): 33-9.
Gilman S, Wenning GK, Low PA, Brooks DJ, Mathias CJ, Trojanowski JQ*, et al.* Second consensus statement on the diagnosis of multiple system atrophy. Neurology 2008; 71(9): 670-6.
Gorno-Tempini ML, Hillis AE, Weintraub S, Kertesz A, Mendez M, Cappa SF*, et al.* Classification of primary progressive aphasia and its variants. Neurology 2011; 76(11): 1006-14.
Hoglinger GU, Respondek G, Stamelou M, Kurz C, Josephs KA, Lang AE*, et al.* Clinical diagnosis of progressive supranuclear palsy: The movement disorder society criteria. Mov Disord 2017; 32(6): 853-64.
Jack CR, Jr., Bennett DA, Blennow K, Carrillo MC, Dunn B, Haeberlein SB*, et al.* NIA-AA Research Framework: Toward a biological definition of Alzheimer's disease. Alzheimers Dement 2018; 14(4): 535-62.

**eMethods 2.** Additional details about [$^{18}$F]RO948 preprocessing

Low-dose CT scans were performed immediately prior to the PET scans for attenuation correction. PET data was reconstructed using VPFX-S (ordered subset expectation maximization combined with corrections for time-of-flight and point spread function), with 6 iterations and 17 subsets with 3 mm smoothing, standard Z filter, and 25.6-cm field of view (256 × 256 matrix). After list-mode data was binned into 4x5-min time frames, PET images were motion corrected (rigid transformation using AFNI, 3dvolreg),[1] summed, and co-registered to their corresponding T1-weighted MR images

**eReference**

1. Cox RW. AFNI: software for analysis and visualization of functional magnetic resonance neuroimages. *Comput Biomed Res.* 1996;29(3):162-173.

**eMethods 3.** Temporal regions of interest in semantic variant primary progressive aphasia

In light of the high white matter signal seen within the temporal lobes of the semantic variant primary progressive aphasia cases that had tau-PET with both [$^{18}$F]flortaucipir and [$^{18}$F]RO948, the use of a composite FreeSurfer based grey matter temporal region of interest (ROI) was felt to be suboptimal. As such, subject specific temporal ROIs were drawn for each case; given the higher retention of [$^{18}$F]flortaucipir, these were initially done on [$^{18}$F]flortaucipir SUVR images and then applied to [$^{18}$F]RO948 SUVR images. After loading the [$^{18}$F]flortaucipir SUVR in PMOD (v.3.7, PMOD Technologies Ltd., Zurich, Switzerland), images were thresholded at 1.05 (i.e. 5% over retention in the reference region, a conservative approach given the reported test-retest findings for both [$^{18}$F]flortaucipir [Devous et al., *J Nucl Med* 2018] and [$^{18}$F]RO948 [Kuwabara et al., *J Nucl Med* 2018]). Using an axial view, voxels with an SUVR above 1.05 were included at each slice. This process began at the very base of the temporal lobe and was repeated until the upper boundary of the middle temporal lobe was reached (no significant retention was noted beyond this area in all three cases).

**eMethods 4.** Additional details about CSF procedures

**CSF collection and analysis**

Lumbar punctures were performed in the morning. CSF samples were collected into 5 ml LoBind polypropylene tubes at the memory clinics in Malmö, Lund and Ängelholm and handled according to the Alzheimer's Association Flow Chart for lumbar puncture.[1] Following centrifugation, 1 ml portions of the supernatant was transferred to 1.5 ml LoBind polypropylene tubes and were frozen at -80° C (within 30 min of collection) pending analyses. The samples were analyzed using commercially available enzyme-linked immunosorbent assays (ELISAs) (INNOTEST, Fujirebio) to determine the levels of $A\beta_{42}$, $A\beta_{40}$ and tau phosphorylated at Thr181 (P-$tau_{181}$). All analyses were performed by board-certified laboratory technicians using procedures accredited by the Swedish Board for Accreditation and Conformity Assessment (SWEDAC) at the Sahlgrenska University Hospital.

**eReference**

[1]Blennow K, Hampel H, Weiner M, Zetterberg H. Cerebrospinal fluid and plasma biomarkers in Alzheimer disease. Nat Rev Neurol. 2010;6(3):131-144.

**eTable 1.** Demographics of young (age 20-40) Aβ-negative controls used to set [$^{18}$F]RO948 SUVR cutoffs

| Characteristic | | Young controls (N=17) |
|---|---|---|
| Age, mean (SD) [range], y | | 30.5 (6.6) [22-39] |
| Sex, Male/Female (% Male) | | 7/10 (59%) |
| Education, y, mean (SD) | | 14.4 (2.4) |
| MMSE score, mean (SD) | | 29.5 (0.87) |
| Aβ status,<br>Neg./ Pos. (% Pos.) | | 17/0 (0%) |
| *APOE* ε4 status<br>Neg./ Pos. (% Pos.) | | 14/3 (18%) |
| [$^{18}$F]RO948, SUVR, mean (SD) in tau-imaging ROIs | | |
| Non-PVC | I-II | 1.06 (0.17) |
| | III-IV | 1.07 (0.12) |
| | I-IV | 1.07 (0.12) |
| | V-VI | 1.05 (0.12) |
| PVC (GTM) | I-II | 1.20 (0.21) |
| | III-IV | 1.15 (0.12) |
| | I-IV | 1.16 (0.12) |
| | V-VI | 1.17 (0.13) |

This age-range was selected in order to ensure a very low likelihood of either amyloid-β or tau pathology.

**eResults .** Participant characteristics

Age differed significantly by group (*F*=8.75, *P*=.003), with AD dementia patients on average being older than controls (*P*<.001), MCI patients (*P*<.05) and patients with non-Alzheimer's disease disorders (*P*<.01). Controls were also younger than patients with MCI or non-Alzheimer's disease disorders (*P*<0.001). There were more males in the non-Alzheimer's disease disorders group compared with the AD dementia (59% vs 43%, *P*<.05) and control (59% vs 46%, *P*<.05) groups. No group differences were seen in years of education across groups. By comparison to controls, MCI and non-Alzheimer's disease patients, MMSE scores were more impaired in AD dementia patients (*F* = 319.2; all post-hoc pairwise comparisons, *P*<.001), with MMSE scores in turn lower among MCI and non-Alzheimer's disease patients as compared to controls (*P*<.001), and among non-Alzheimer's disease patients as compared to MCI (*P*<.001). Rates of amyloid-β positivity (controls, 37%; MCI; 62%; non-Alzheimer's disease, 41% and Alzheimer's disease dementia, 100% [by design]) and *APOE* ε4 carriership (controls, 44%; MCI; 54%; non-Alzheimer's disease, 34% and Alzheimer's disease dementia, 90%), were in line with literature-based prevalence estimates. For [$^{18}$F]RO948 ROIs, mean SUVR values were higher among Alzheimer's disease dementia patients as compared to all groups (*F* range: 147.9-207.2; all post-hoc pairwise comparisons *P*<.001), and higher in MCI subjects compared to controls (*P*<.001) and non-Alzheimer's disease patients (*P*<.01) across I-II, III-IV and I-IV tau-imaging ROIs. Among non-Alzheimer's disease patients, [$^{18}$F]RO948 SUVR values were highest among DLB patients and lowest in those with MSA.

**eTable 2.** Demographics for non-Alzheimer disease subgroups

| Characteristic | BvFTD (N=12) | SvPPA (N=7) | DLB (N=25) | PSP (N=16) | MSA (N=6) | PD/PDD (N=26) | VaD (N=10) |
|---|---|---|---|---|---|---|---|
| Age, y, mean (SD) [range] | 67.3 (9.3) [56, 83] | 71.4 (8.7) [59, 82] | 74 (6.1)[a,g,j] [63, 84] | 66.9 (8.31) [49, 78] | 65.8 (4.8) [60, 73] | 70.3 (10) [36, 85] | 74.8 (7.5)[j] [65, 87] |
| Sex, Male/Female (% Male) | 4/8 (33%) | 3/4 (57%) | 20/5 (80%)[g] | 7/9 (44%) | 5/1 (67%) | 16/10 (62%) | 6/4 (60%) |
| Education, y, mean (SD) | 11 (2.7) | 12 (3.4) | 12.2 (3.8) | 13.1 (3.4) | 11.5 (2.7) | 13.7 (4) | 11.3 (2.8) |
| MMSE score, mean (SD) | 24.6 (2.7)[c] | 21.7 (6.2)[c,e] | 22.4 (5.4)[h,k] | 25.9 (3.6) | 26.3 (3.7) | 27.8 (3.4)[r] | 23.9 (3.9)[d] |
| Aβ status, Neg./ Pos. (% Pos.) | 6/6 (50%) | 2/5 (71%) | 10/15 (60%)[m] | 10/6 (38%)[q] | 4/2 (33%) | 20/6 (23%) | 7/3 (30%) |
| *APOE* ε4 status Neg./ Pos. (% Pos.) | 8/4 (33%) | 6/1 (17%) | 12/13 (52%) | 11/5 (31%) | 4/2 (33%) | 19/7 (27%) | 8/2 (20%) |
| [18F]RO948 SUVR, mean (SD) within tau-imaging ROIs | | | | | | | |
| I-II | 1.30 (0.67) | 1.22 (0.19) | 1.40 (0.34)[i,j,l] | 1.14 (0.27) | 1.09 (0.09) | 1.18 (0.23) | 1.20 (0.21) |
| III-IV | 1.18 (0.23) | 1.22 (0.07)[f] | 1.31 (0.22)[a,i,j,m,p] | 1.22 (0.31) | 1.14 (0.08) | 1.17 (0.12) | 1.10 (0.10) |
| I-IV | 1.18 (0.24) | 1.22 (0.08)[f] | 1.31 (0.23)[a.i.j,m,p] | 1.21 (0.31) | 1.13 (0.08) | 1.17 (0.13) | 1.10 (0.10) |
| V-VI | 1.01 (0.13) | 1.02 (0.06) | 1.10 (0.12)[o] | 1.09 (0.22) | 1.06 (0.08) | 1.05 (0.11) | 1.00 (0.11) |

BvFTD, behavioral variant frontotemporal dementia; SvPPA, semantic variant primary progressive aphasia; DLB, dementia with Lewy bodies; PSP, progressive supranuclear palsy; MSA, multiple system atrophy; PD/PDD, Parkinson's disease without and with dementia; VaD, vascular dementia. [a] Significantly higher than bvFTD, $P<.05$; [b] Significantly higher than DLB, $P<.05$; [c] Significantly lower than PD/PDD, $P<.001$;

[d] Significantly lower than bvFTD, $P<.05$; [e] Significantly lower than MSA, $P<.001$; [f] Significantly higher than MSA, $P<.05$; [g] Significantly higher than PSP, $P<.05$; [h] Significantly lower than PSP, $P<.01$; [i] Significantly higher than PSP, $P<.001$; [j] Significantly higher than MSA, $P<.05$; [k] Significantly lower than PD/PDD, $P<.001$; [l] Significantly higher than PD/PDD, $P<.05$; [m] Significantly higher than PD/PDD, $P<.01$; [o] Significantly higher than VaD, $P<.05$; [p] Significantly higher than VaD, $P<.01$; [q] Significantly higher than VaD, $P<.001$; [r] Significantly lower than VaD, $P<.01$.

**eTable 3**. Partial volume error corrected [$^{18}$F]RO948 SUVR data for cognitively unimpaired controls and mild cognitive impairment, Alzheimer disease dementia and non-Alzheimer disease disorder patients

| Characteristic | CU controls (N=257) | MCI (N=154) | AD dementia (N=100) | Non-AD (N=102) |
|---|---|---|---|---|
| [$^{18}$F]RO948 SUVR, mean (SD) within tau-imaging ROIs | | | | |
| I-II | 1.33 (0.30) | 1.61 (0.50)[b,d] | 2.52 (0.55)[b,c,e] | 1.47 (0.53)[a] |
| III-IV | 1.28 (0.19) | 1.47 (0.43)[b] | 2.73 (0.10)[b,c,e] | 1.39 (0.28)[b] |
| I-IV | 1.28 (0.18) | 1.47 (0.43)[b] | 2.71 (0.97)[b,c,e] | 1.39 (0.29)[b] |
| V-VI | 1.21 (0.13) | 1.27 (0.21) | 1.91 (0.63)[b,c,e] | 1.25 (0.18) |

CU, cognitively unimpaired; MCI, mild cognitive impairment; AD dementia, dementia due to Alzheimer's disease; Non-AD, non-Alzheimer's disease neurodegenerative disorders. [a] Significantly higher than CU controls, $P<.05$; [b] Significantly higher than CU controls, $P<.001$; [c] Significantly higher than MCI, $P<.001$; [d] Significantly higher than non-AD, $P<.01$; [e] Significantly higher than non-AD, $P<.001$.

**eTable 4.** Partial volume error corrected [$^{18}$F]RO948 SUVR data for non-AD subgroups

| Characteristic | BvFTD (N=12) | SvPPA (N=7) | DLB (N=25) | PSP (N=16) | MSA (N=6) | PD/PDD (N=26) | VaD (N=10) |
|---|---|---|---|---|---|---|---|
| [$^{18}$F]RO948 SUVR, mean (SD) within tau-imaging ROIs | | | | | | | |
| I-II | 1.38 (0.33) | 1.58 (0.31)[b,d,f] | 1.71 (0.39)[c,e,g,h] | 1.26 (0.24) | 1.20 (0.10) | 1.37 (0.35) | 1.39 (0.42) |
| III-IV | 1.36 (0.30) | 1.48 (0.11)[a,c,d,g,h] | 1.59 (0.42) [c,e,g,h] | 1.31 (0.17) | 1.25 (0.09) | 1.32 (0.15) | 1.27 (0.12) |
| I-IV | 1.37 (0.32) | 1.48 (0.11)[a,c,d,g,h] | 1.59 (0.42) [c,e,g,h] | 1.30 (0.17) | 1.24 (0.09) | 1.32 (0.16) | 1.27 (0.11) |
| V-VI | 1.20 (0.15) | 1.22 (0.08) | 1.35 (0.28)b,h | 1.23 (0.08) | 1.20 (0.10) | 1.24 (0.14) | 1.17 (0.13) |

BvFTD, behavioral variant frontotemporal dementia; SvPPA, semantic variant primary progressive aphasia; DLB, dementia with Lewy bodies; PSP, progressive supranuclear palsy; MSA, multiple system atrophy; PD/PDD, Parkinson's disease without and with dementia; VaD, vascular dementia. [a] Significantly higher than bvFTD, $P<.05$; [b] Significantly higher than PSP, $P<.05$; [c] Significantly higher than PSP, $P<.01$; [d] Significantly higher than MSA, $P<.05$; [e] Significantly higher than MSA, $P<.01$; [f] Significantly higher than PD/PDD, $P<.05$; [g] Significantly higher than PD/PDD, $P<.01$; [h] Significantly higher than VaD, $P<.01$.

**eTable 5.** Demographics for svPPA cases

| Case | Age | Sex | Education | MMSE | *APOE* | Aβ-status |
|---|---|---|---|---|---|---|
| 1 | 59.6 | F | 14 | 26 | ε3/ ε3 | Negative |
| 2 | 82.1 | F | 7 | 18 | ε3/ ε3 | Positive |
| 3 | 75.2 | F | 12 | 27 | ε4/ ε3 | Negative |

SvPPA, semantic variant primary progressive aphasia; Aβ-status was based on the CSF $A\beta_{42}/A\beta_{42}$, using a cutoff of $< 0.089$.

**eTable 6.** Diagnostic performance of [$^{18}$F]RO948 SUVR using the tau-imaging I-IV ROI for AD dementia and Aβ-positive MCI versus non-AD disorders

| | **AUC (95% CI)** | **Agreement % (95% CI)** | **Specificity % (95% CI)** | **Positive Likelihood ratio (95% CI)** | **Negative Likelihood ratio (95% CI)** |
|---|---|---|---|---|---|
| **AD dementia (n=100) (Sensitivity 92.0% [95% CI, 84.8%, 96.5%])** | | | | | |
| *vs* Cognitively unimpaired controls | 0.98 (0.96-0.99) | 93.9 (91.4, 96.4) | 95.1 (92.4, 97.3) | 17.20 (10.10, 29.31) | 0.10 (0.05, 0.18) |
| *vs* All non-AD disorders | 0.97 (0.95, 0.99) | 91.3 (87.2, 94.9) | 90.6 (84.4, 95.8) | 8.73 (4.84, 15.74) | 0.10 (0.05, 0.19) |
| *vs* All Aβ-positive non-AD disorders | 94.0 (91.0, 98.0) | 88.0 (82.4, 93.0) | 76.0 (60.0, 89.2) | 2.41 (1.70, 3.42) | 2.41 (1.70, 3.42) |
| *vs* All Aβ-negative non-AD disorders | 0.99 (0.97, 1.00) | 95.0 (91.1, 98.1) | 100 (100, 100) | 3.59 (2.45, 5.27) | 0.12 (0.06, 0.23) |
| *vs* Multiple system atrophy | 0.99 (0.98, 1.00) | 92.3 (86.4, 97.1) | 100 (100, 100) | NC | 0.09 (0.05, 0.17) |
| *vs* Frontotemporal dementia disorders | 0.98 (0.96, 1.00) | 93.2 (88.0, 97.4) | 100 (100, 100) | NC | 0.09 (0.05, 0.17) |
| *vs* Behavioural variant frontotemporal dementia | 0.99 (0.97, 1.00) | 93.0 (87.3, 97.3) | 100 (100, 100) | NC | 0.09 (0.05, 0.17) |

| | | | | | |
|---|---|---|---|---|---|
| *vs* Semantic variant primary progressive aphasia | 0.97 (0.95, 1.00) | 93.0 (87.0, 97.2) | 100 (100, 100) | NC | 0.09 (0.05, 0.17) |
| *vs* Movement disorders | 0.96 (0.94, 0.99) | 90.0 (85.0, 94.0) | 86.0 (76.2, 94.0) | 0.11 (0.06, 0.20) | 0.11 (0.06, 0.20) |
| *vs* Progressive supranuclear palsy | 0.98 (0.95, 1.00) | 92.0 (86.7, 96.5) | 93.0 (79.0, 100) | 14.55 (2.18, 97.13) | 0.10 (0.05, 0.18) |
| *vs* Dementia with Lewy bodies | 0.93 (0.88, 0.97) | 88.0 (82.0, 93.4) | 70.0 (52.3, 87.0) | 2.84 (1.60, 5.05) | 0.13 (0.07, 0.26) |
| *vs* Parkinson's disease | 0.98 (0.96, 1.00) | 93.0 (88.0, 97.0) | 96.2 (86.0, 100) | 23.64 (3.46, 161.69) | 0.09 (0.05, 0.18) |
| **Aβ-positive MCI (n=96) (Sensitivity 35.4% [95% CI, 25.9%, 45.8%])** | | | | | |
| *vs* Cognitively unimpaired controls | 0.78 (0.72, 0.84) | 80.6 (77.3, 83.9) | 92.6 (89.6, 95.6) | 12.22 (7.06, 21.17) | 0.37 (0.28, 0.49) |
| *vs* All non-AD disorders | 0.73 (0.66, 0.80) | 65.0 (59.0, 70.1) | 90.1 (84.2, 95.1) | 6.20 (3.39, 11.35) | 0.40 (0.30, 0.52) |
| *vs* All Aβ-positive non-AD disorders | 0.59 (0.48, 0.69) | 49.0 (41.2, 57.0) | 75.0 (63.0, 88.0) | 1.71 (1.17, 2.49) | 0.57 (0.40, 0.80) |
| *vs* All Aβ-negative non-AD disorders | 0.75 (0.68, 0.83) | 62.0 (56.1, 68.0) | 100 (100, 100) | NC | 0.35 (0.27, 0.46) |
| *vs* Multiple system atrophy | 0.77 (0.63, 0.93) | 0.41 (0.32, 0.50) | 100 (100, 100) | NC | 0.35 (0.27, 0.46) |

| | | | | | |
|---|---|---|---|---|---|
| *vs* Frontotemporal dementia disorders | 0.70 (0.59, 0.81) | 47.4 (40.0, 56.1) | 100 (100, 100) | NC | 0.35 (0.27, 0.46) |
| *vs* Behavioural variant frontotemporal dementia | 0.77 (0.65, 0.89) | 44.0 (36.0, 52.3) | 100 (100, 100) | NC | 0.35 (0.27, 0.46) |
| *vs* Semantic variant primary progressive aphasia | 0.60 (0.45-0.75) | 42.0 (33.0, 51.0) | 100 (100, 100) | NC | 0.35 (0.27, 0.46) |
| *vs* Movement disorders | 0.66 (0.58, 0.74) | 57.1 (51.0, 64.0) | 85.1 (76.1, 93.0) | 4.33 (2.40, 7.81) | 0.42 (0.31, 0.56) |
| *vs* Progressive supranuclear palsy | 0.75 (0.63, 0.87) | 46.0 (37.0, 54.0) | 94.0 (81.0, 100) | 10.33 (1.54, 69.33) | 0.38 (0.28, 0.51) |
| *vs* Dementia with Lewy bodies | 0.48 (0.35, 0.61) | 58.0 (50.0, 66.1) | 68.0 (48.0, 84.0) | 2.02 (1.12, 3.64) | 0.52 (0.36, 0.76) |
| *vs* Parkinson’s disease | 0.74 (0.64, 0.84) | 50.0 (42.0, 58.2) | 96.2 (86.0, 100) | 16.79 (2.44, 115.41) | 0.37 (0.28, 0.49) |

NC, not calculated due 100% specificity (Positive likelihood ratio = Sensitivity/(1-Specificity).

Frontotemporal dementia disorders include behavioural variant frontotemporal dementia and semantic variant primary progressive aphasia. Movement disorders includes Parkinson’s with and without dementia, dementia with Lewy bodies, and progressive supranuclear palsy.

**eTable 7.** Diagnostic performance of [$^{18}$F]RO948 SUVR using individual tau imaging ROIs

| Tau-imaging ROI | Cutoff | AUC (95% CI) | Agreement (95% CI) | Sensitivity (95% CI) | Specificity (95% CI) | Positive Likelihood ratio (95% CI) | Negative Likelihood ratio (95% CI) |
|---|---|---|---|---|---|---|---|
| **AD dementia (n=100) *vs* CU controls (n=257)** | | | | | | | |
| I-II | > 1.48 | 0.97 (0.95-0.98) | 92.3 (89.2, 94.8) | 91.9 (85.9, 97.0) | 92.4 (88.9, 95.4) | 11.31 (7.40, 17.28) | 0.09 (0.05, 0.17) |
| III | > 1.29 | 0.97 (0.95-0.99) | 92.8 (90.8, 95.3) | 91.9 (85.9, 97.0) | 93.2 (90.1, 95.8) | 12.99 (8.29, 20.37) | 0.10 (0.05, 0.18) |
| IV | > 1.41 | 0.96 (0.94-0.98) | 92.8 (90.1, 95.3) | 82.8 (74.7, 89.9) | 96.6 (94.3, 98.5) | 23.42 (12.24, 44.78) | 0.19 (0.12, 0.28) |
| V | > 1.38 | 0.91 (0.87-0.95) | 89.2 (86.2, 92.0) | 65.0 (56.0, 74.0) | 98.5 (97.0, 99.6) | 53.76 (17.29, 167.16) | 0.36 (0.28, 0.47) |
| VI | > 1.33 | 0.76 (0.71-0.83) | 81.0 (78.2, 83.1) | 30.3 (21.2, 39.4) | 100 (100, 100) | NC | 0.70 (0.62, 0.80) |
| **AD dementia (n=100) *vs* non-AD disorders (n=102)** | | | | | | | |
| I-II | > 1.48 | 0.96 (0.93, 0.99) | 90.3 (86.1, 94.4) | 92.9 (87.9, 97.0) | 87.5 (80.0, 93.8) | 6.79 (4.08, 11.29) | 0.09 (0.05, 0.18) |
| III | > 1.29 | 0.96 (0.94, 0.99) | 90.8 (86.7, 94.4) | 92.9 (87.9, 98.0) | 88.5 (82.3, 94.8) | 7.93 (4.53, 13.88) | 0.10 (0.05, 0.19) |

| | | | | | | | |
|---|---|---|---|---|---|---|---|
| IV | > 1.41 | 0.95 (0.92, 0.98) | 87.7 (83.1, 92.0) | 83.8 (75.8, 91.0) | 91.7 (85.4, 97.0) | 9.82 (5.02, 19.19) | 0.20 (0.13, 0.30) |
| V | > 1.38 | 91.7 (87.7, 96.0) | 81.5 (76.4, 86.2) | 65.0 (56.0, 74.0) | 99.0 (97.0, 100) | 30.86 (7.77, 122.57) | 0.36 (0.28, 0.48) |
| VI | > 1.33 | 79.4 (73.1, 85.6) | 64.1 (60.0, 68.7) | 30.3 (21.2, 39.4) | 99.0 (96.9, 100) | 29.09 (4.05, 209.12) | 0.70 (0.62, 0.80) |
| **Aβ-positive MCI (n=96) *vs* CU controls (n=257)** | | | | | | | |
| I-II | > 1.48 | 0.78 (0.72, 0.84) | 80.6 (77.3, 83.9) | 46.9 (37.5, 56.3) | 92.6 (89.6, 95.6) | 5.77 (3.60, 9.23) | 0.58 (0.48, 0.70) |
| III | > 1.29 | 0.79 (0.76, 0.83) | 79.2 (75.7, 82.5) | 39.6 (30.2, 50.0) | 93.3 (90.4, 96.3) | 5.54 (3.33, 9.22) | 0.65 (0.55, 0.77) |
| IV | > 1.41 | 0.72 (0.65, 0.78) | 79.0 (76.2, 82.0) | 29.2 (20.1, 38.5) | 96.7 (94.3, 98.5) | 8.17 (4.00, 16.67) | 0.73 (0.64, 0.84) |
| V | > 1.38 | 0.58 (0.51, 0.65) | 76.5 (74.3, 78.7) | 14.6 (8.3, 21.9) | 98.5 (97.0, 100) | 11.96 (3.51, 40.69) | 0.86 (0.80, 0.94) |
| VI | > 1.33 | 0.50 (0.43, 0.57) | 74.0 (73.0, 75.0) | 1.04 (0, 3.1) | 100 (100, 100) | NC | 0.99 (0.97, 1.01) |
| **Aβ-positive MCI (n=96) *vs* non-AD disorders (n=102)** | | | | | | | |
| I-II | > 1.48 | 0.72 (0.65, 0.79) | 68.0 (61.4, 73.6) | 47.0 (37.1, 57.3) | 87.1 (80.2, 94.1) | 3.46 (2.00, 5.99) | 0.61 (0.50, 0.75) |

| | | | | | | | |
|---|---|---|---|---|---|---|---|
| III | > 1.29 | 0.70 (0.65-0.79) | 63.7 (58.0, 69.4) | 39.2 (28.9, 50.0) | 88.5 (82.3, 94.8) | 3.45 (1.88, 6.35) | 0.68 (0.57, 0.81) |
| IV | > 1.41 | 0.65 (0.57-0.73) | 60.1 (54.9, 65.8) | 28.9 (20.0, 38.1) | 91.7 (87.0, 97.0) | 3.72 (1.78, 7.75) | 0.77 (0.67, 0.88) |
| V | > 1.38 | 0.59 (0.51-0.67) | 56.5 (53.0, 60.1) | 14.4 (8.3, 21.6) | 99.0 (97.0, 100) | 7.00 (1.63, 29.97) | 0.87 (0.80, 0.95) |
| VI | > 1.33 | 0.56 (0.48-0.64) | 50.0 (48.2, 51.3) | 1.03 (0, 3.1) | 99.0 (97.0, 100) | 1.06 (0.07, 16.75) | 1.00 (0.97, 1.03) |

NC, not calculated due 100% specificity (Positive likelihood ratio = Sensitivity/(1-Specificity). By comparison to Table 2, where diagnostic performance of [$^{18}$F]RO948 PET was assessed using I-II, III-IV, I-V and V-VI ROIs, the use of individual ROIs (I-II, III, IV, V, and VI) resulted in somewhat lower AUCs when using the stage VI ROI for contrasts involving AD dementia, and somewhat lower AUCs when using the stage IV ROI for contrasts involving Aβ-positive MCI.

**eTable 8.** AUC values for [$^{18}$F]RO948 SUVR using different cutoffs (based on mean+2.5, 2 and 1.5 SD in Aβ-negative young controls)

| | **Cutoff using Mean+2.5 SD** | | **Cutoff using Mean+2 SD** | | **Cutoff using Mean+1.5 SD** | |
|---|---|---|---|---|---|---|
| **Tau-imaging ROI** | **Sensitivity** | **Specificity** | **Sensitivity** | **Specificity** | **Sensitivity** | **Specificity** |
| **AD dementia (n=100) *vs* CU controls (n=257)** | | | | | | |
| I-II | 91.9 (85.9, 97.0) | 92.4 (88.9, 95.4) | 94.9 (89.9, 99.0) | 90.5(86.7, 93.9) | 97.0 (92.9, 100) | 85.6 (81.0, 89.4) |
| I-IV | 91.0 (84.9, 96.0) | 95.1 (92.4, 97.3) | 96.0 (90.9, 99.0) | 89.4 (85.6, 92.8) | 99.0 (97.0, 100) | 76.8 (71.9, 82.1) |
| V-VI | 59.6 (49.9, 69.7) | 98.5 (97.0, 99.6) | 64.6 (54.5, 73.7) | 98.1 (96.6, 99.6) | 70.7 (61.6, 78.8) | 96.2 (93.5, 98.5) |
| **AD dementia (n=100) *vs* non-AD disorders (n=102)** | | | | | | |
| I-II | 92.9 (87.9, 97.0) | 87.5 (80.0, 93.8) | 96.0 (91.9, 99.0) | 83.3 (76.0, 90.6) | 98.0 (94.9, 100) | 76.0 (67.7, 84.4) |
| I-IV | 91.9 (85.9, 97.0) | 90.6 (84.4, 95.8) | 96.0 (91.9, 99.0) | 82.3 (75.0, 89.6) | 99.0 (97.0, 100) | 63.5 (53.1, 72.9) |
| V-VI | 59.6 (50.5, 69.7) | 97.9 (94.8, 100) | 64.6 (54.5, 74.7) | 97.9 (94.8, 100) | 70.7 (61.6, 79.8) | 97.9 (94.8, 100) |
| **Aβ-positive MCI (n=96) *vs* CU controls (n=257)** | | | | | | |
| I-II | 46.9 (37.5, 56.3) | 92.6 (89.6, 95.6) | 50.5 (40.2, 60.8) | 83.3 (75.0, 90.6) | 58.8 (49.5, 68.0) | 76.0 (66.7, 84.4) |

| | | | | | | |
|---|---|---|---|---|---|---|
| I-IV | 37.5 (28.1, 47.0) | 95.2 (92.6, 97.8) | 47.4 (37.1, 57.7) | 82.3 (74.0, 89.6) | 59.8 (49.5, 69.1) | 63.5 (53.1, 72.9) |
| V-VI | 13.00 (6.3, 18.8) | 99.0 (97.0, 100) | 15.5 (9.3, 22.7) | 97.9 (94.8, 100) | 17.5 (10.3 25.8) | 97.9 (94.8, 100) |
| **Aβ-positive MCI (n=96) *vs* non-AD disorders (n=102)** | | | | | | |
| I-II | 47.0 (37.1, 57.3) | 87.1 (80.2, 94.1) | 50.5 (40.2, 60.8) | 83.3 (76.0, 90.6) | 58.8 (49.5, 68.0) | 76.0 (66.7, 84.4) |
| I-IV | 37.5 (28.1, 46.9) | 90.1 (84.2, 95.1) | 47.4 (37.1, 56.7) | 82.3 (74.0, 89.6) | 59.8 (49.5, 70.1) | 63.5 (54.2, 72.9) |
| V-VI | 13.00 (6.3, 19.8) | 97 (94.1, 100) | 15.5 (8.3, 22.7) | 97.9 (94.8, 100) | 17.5 (10.3, 24.7) | 97.9 (94.8, 100) |

Cutoffs for [$^{18}$F]RO948 SUVR across tau-imaging ROIs were as follows (mean+2.5SD, 2SD and 1.5SD: I-II, 1.48, 1.41, and 1.32; I-IV, 1.36, 1.28, and 1.22; V-VI, 1.35, 1.30, 1.24).

**eTable 9.** Diagnostic performance of CSF $A\beta_{42}/A\beta_{40}$ for AD dementia and Aβ positive MCI versus other non-AD disorders and CU controls

| | **Cutoff** | **AUC (95% CI)** | **Agreement (95% CI)** | **Sensitivity (95% CI)** | **Specificity (95% CI)** | **Positive Likelihood ratio (95% CI)** | **Negative Likelihood ratio (95% CI)** |
|---|---|---|---|---|---|---|---|
| **AD dementia (n=100) vs CU controls (n=257)** | | | | | | | |
| CSF $A\beta_{42}/A\beta_{40}$ | < 0.089 | 0.91 (0.88, 0.94) | 73.8 (69.6, 78.2) | 100 (100, 100) | 63.9 (58.2, 69.6) | 2.60 (2.22, 3.04) | NC |
| **AD dementia (n=100) vs non-AD disorders (n=102)** | | | | | | | |
| CSF $A\beta_{42}/A\beta_{40}$ | < 0.089 | 0.93 (0.89, 0.96) | 81.0 (76.4, 85.6) | 100 (100, 100) | 61.5 (52.1, 70.8) | 2.43 (1.93, 3.06) | NC |
| **Aβ-positive MCI (n=96) vs CU controls (n=257)** | | | | | | | |
| CSF $A\beta_{42}/A\beta_{40}$ | < 0.089 | 0.89 (0.84-0.93) | 62.5 (53.1, 71.9) | 100 (100, 100) | 63.8 (57.7, 69.4) | 2.62 (2.24, 3.06) | NC |
| **Aβ-positive MCI (n=96) vs non-AD disorders (n=102)** | | | | | | | |
| CSF $A\beta_{42}/A\beta_{40}$ | < 0.089 | 0.89 (0.84, 0.93) | 81.5 (76.7, 86.0) | 100 (100, 100) | 62.5 (52.1, 72.0) | 2.43 (1.93, 3.06) | NC |

NC, not calculated due 100% sensitivity (Negative likelihood ratio = (1-Sensitivity)/Specificity.

**eTable 10.** [$^{18}$F]Flortaucipir and [$^{18}$F]RO948 SUVR values in temporal and primary somatosensory cortex ROIs for semantic variant primary progressive aphasia

| | Temporal ROI | | | Primary somatosensory cortex ROI | | Temporal > Somatosensory, % Diff. | |
|---|---|---|---|---|---|---|---|
| Case | [$^{18}$F]Flortaucipir | [$^{18}$F]RO948 | % Diff. temporal ROI | [$^{18}$F]Flortaucipir | [$^{18}$F]RO948 | [$^{18}$F]Flortaucipir | [$^{18}$F]RO948 |
| 1 | 1.71 | 1.36 | 23 | 0.98 | 0.94 | 54 | 37 |
| 2 | 1.92 | 1.48 | 27 | 0.88 | 0.90 | 74 | 49 |
| 3 | 1.42 | 1.20 | 17 | 0.86 | 0.88 | 49 | 31 |

**eTable 11.** Area under the receiver operating characteristic curve values for [$^{18}$F]RO948 SUVR in tau imaging ROIs with and without subjects showing high skull/meningeal signal

| | AUC (95% CI) | |
|---|---|---|
| Tau-imaging ROI | All subjects | High skull/meningeal excluded |
| **AD dementia *vs* CU controls** | | |
| I-II | 0.973 (0.951-0.983) | 0.975 (0.951-0.983) |
| III-IV | 0.970 (0.953-0.987) | 0.972 (0.956-0.989) |
| I-IV | 0.977 (0.961-0.993) | 0.978 (0.962-0.994) |
| V-VI | 0.910 (0.871-0.949) | 0.913 (0.873-0.952) |
| **AD dementia *vs* non-AD disorders** | | |
| I-II | 0.964 (0.935, 0.986) | 0.961 (0.935-0.987) |
| III-IV | 0.961 (0.938, 0.984) | 0.963 (0.940-0.986) |
| I-IV | 0.971 (0.950, 0.991) | 0.971 (0.950-0.992) |
| V-VI | 0.919 (0.881, 0.958) | 0.922 (0.883-0.961) |
| **Aβ-positive MCI *vs* CU controls** | | |
| I-II | 0.776 (0.722, 0.838) | 0.794 (0.737-0.852) |
| III-IV | 0.772 (0.673, 0.802) | 0.793 (0.696-0.835) |
| I-IV | 0.797 (0.753-0.854) | 0.816 (0.740-0.851) |
| V-VI | 0.585 (0.517, 0.656) | 0.606 (0.534-0.678) |
| **Aβ-positive MCI *vs* non-AD disorders** | | |
| I-II | 0.724 (0.647, 0.788) | 0.736 (0.665-0.807) |
| III-IV | 0.705 (0.635, 0.776) | 0.720 (0.646-0.784) |
| I-IV | 0.729 (0.655, 0.796) | 0.734 (0.676-0.804) |
| V-VI | 0.606 (0.526, 0.685) | 0.618 (0.605-0.708) |

27 subjects (4.4%) were found to have elevated retention of [$^{18}$F]R0948 in the skull/meninges, defined as confluent signal upon visual inspection with SUVR>2.5: four AD dementia, 14 CU

(10 Aβ-negative, four Aβ-positive), four Aβ-positive MCI, and five non-AD (two bvFTD, one PSP, one PD/PDD and one VaD). Excluding these cases from the original (all subject) analyses did not result in any significant changes in ROC derived AUC values for [$^{18}$F]RO948 PET SUVR in tau-imaging ROIs. Findings are here reported to three decimal points in order to facilitate comparison.

**eFigure 1.** Tau PET imaging composite ROIs approximating the Braak post-mortem staging scheme for tau pathology

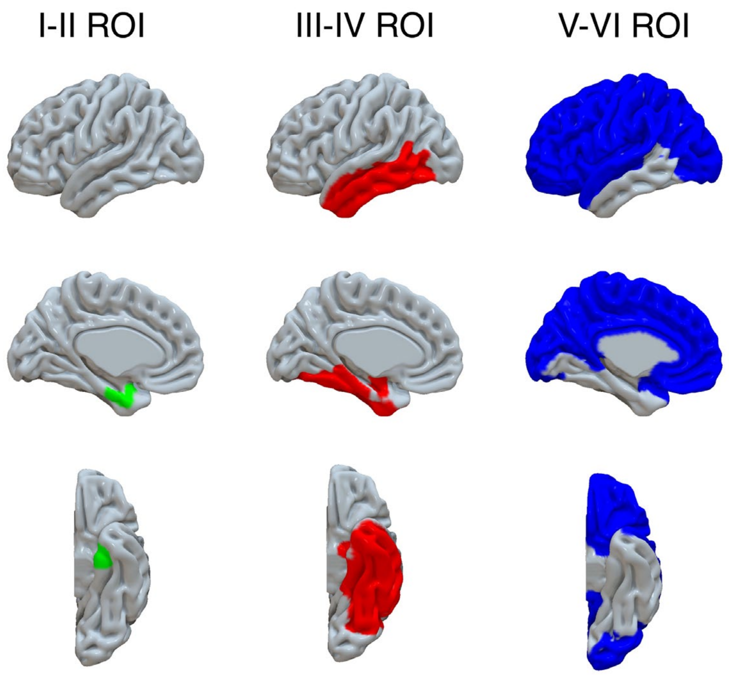


Top, middle and bottom rows show dorsal, medial and inferior views of the left hemisphere (for illustrative purposes; ROIs bilateral).

*I–II ROI*: entorhinal cortex; *III-IV ROI*: amygdala, fusiform gyrus, inferior temporal cortex, middle temporal cortex, and parahippocampus; *V-V ROII*: anterior cingulate, inferior frontal cortex, inferior parietal cortex, insular cortex, lateral occipital cortex, lingual gyrus, medial occipital cortex, middle frontal cortex, orbitofrontal cortex, paracentral cortex, precentral cortex, precuneus, postcentral cortex, posterior cingulate, superior frontal cortex, superior parietal cortex, superior temporal gyrus, and supramarginal gyrus.

**eFigure 2.** Mean [$^{18}$F]RO948 images and scatterplots for the young (age 20-40) Aβ-negative controls used to set cutoffs for [$^{18}$F]RO948 SUVR across tau-imaging ROIs

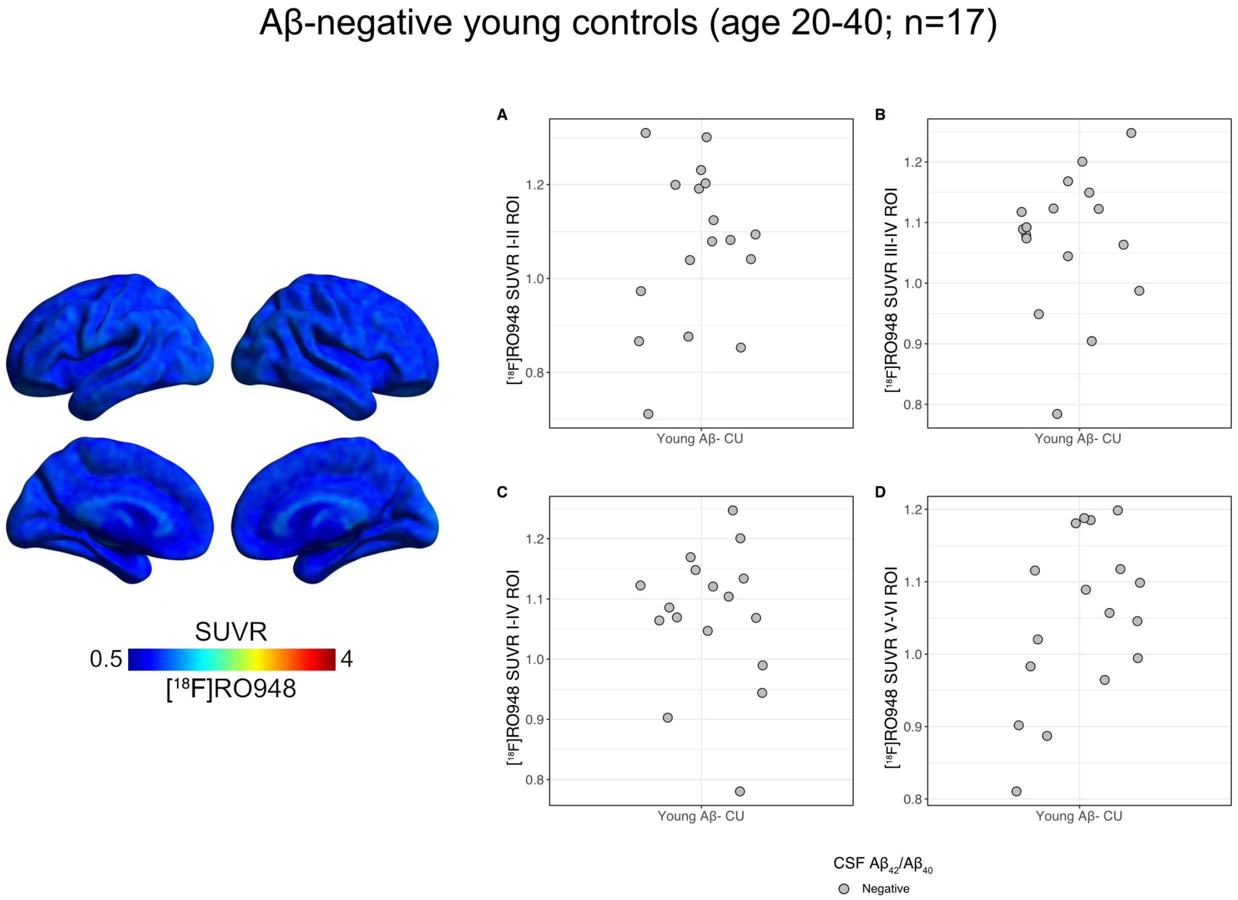

**eFigure 3.** Voxelwise group differences in [$^{18}$F]RO948 SUVR

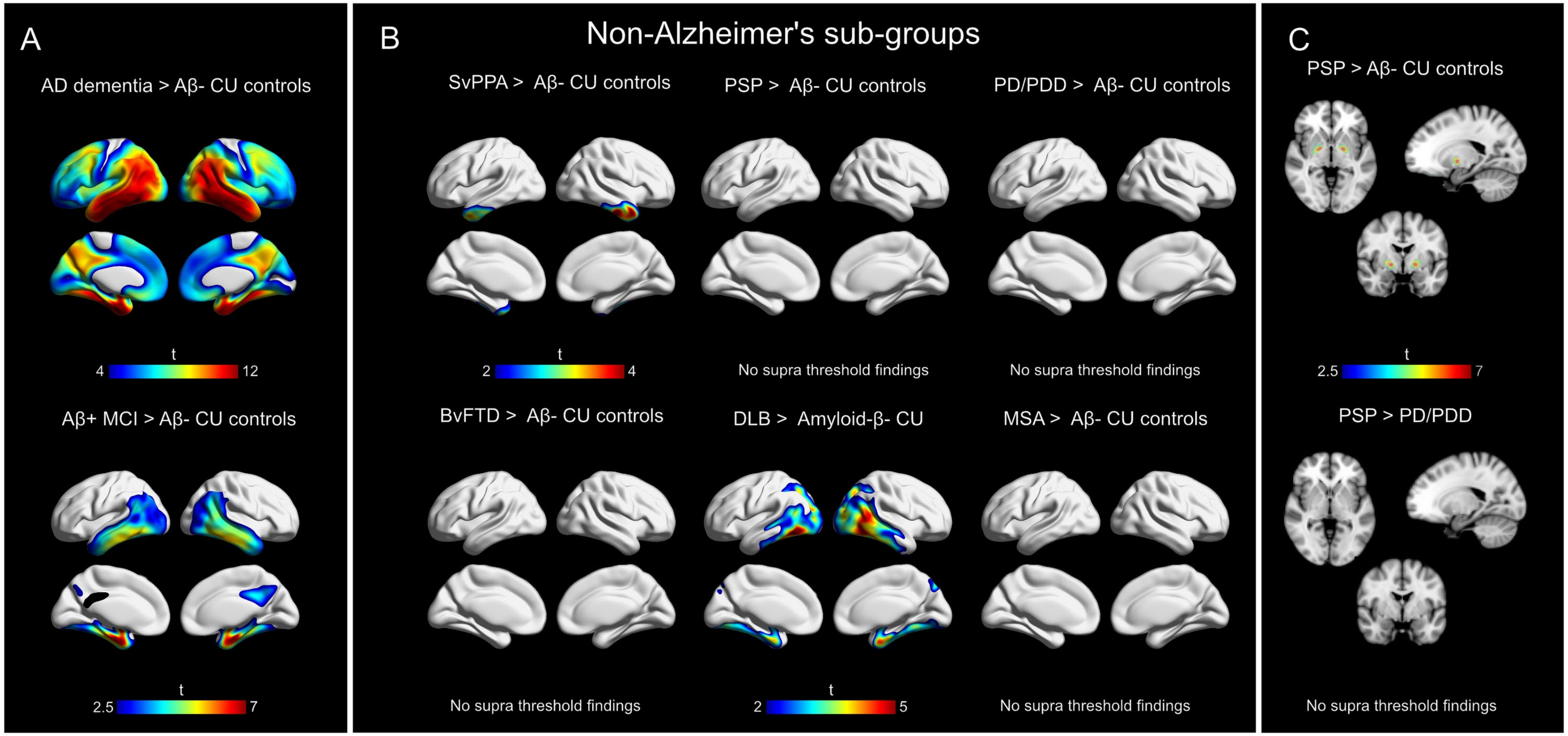


(A) Alzheimer's disease dementia greater than Aβ-negative cognitively unimpaired (CU) controls and Aβ-positive mild cognitive impairment. (B) Non-Alzheimer's disease sub-groups as compared Aβ-negative CU controls. (C) Progressive supranuclear palsy greater than Aβ-negative cognitively unimpaired (CU) controls and Parkinson's disease. A cluster threshold of 100 voxels was applied, with no correction for multiple comparisons (*P*<.001).

**eFigure 4.** Voxelwise group differences in [$^{18}$F]RO948 SUVR using family wise error corrected data

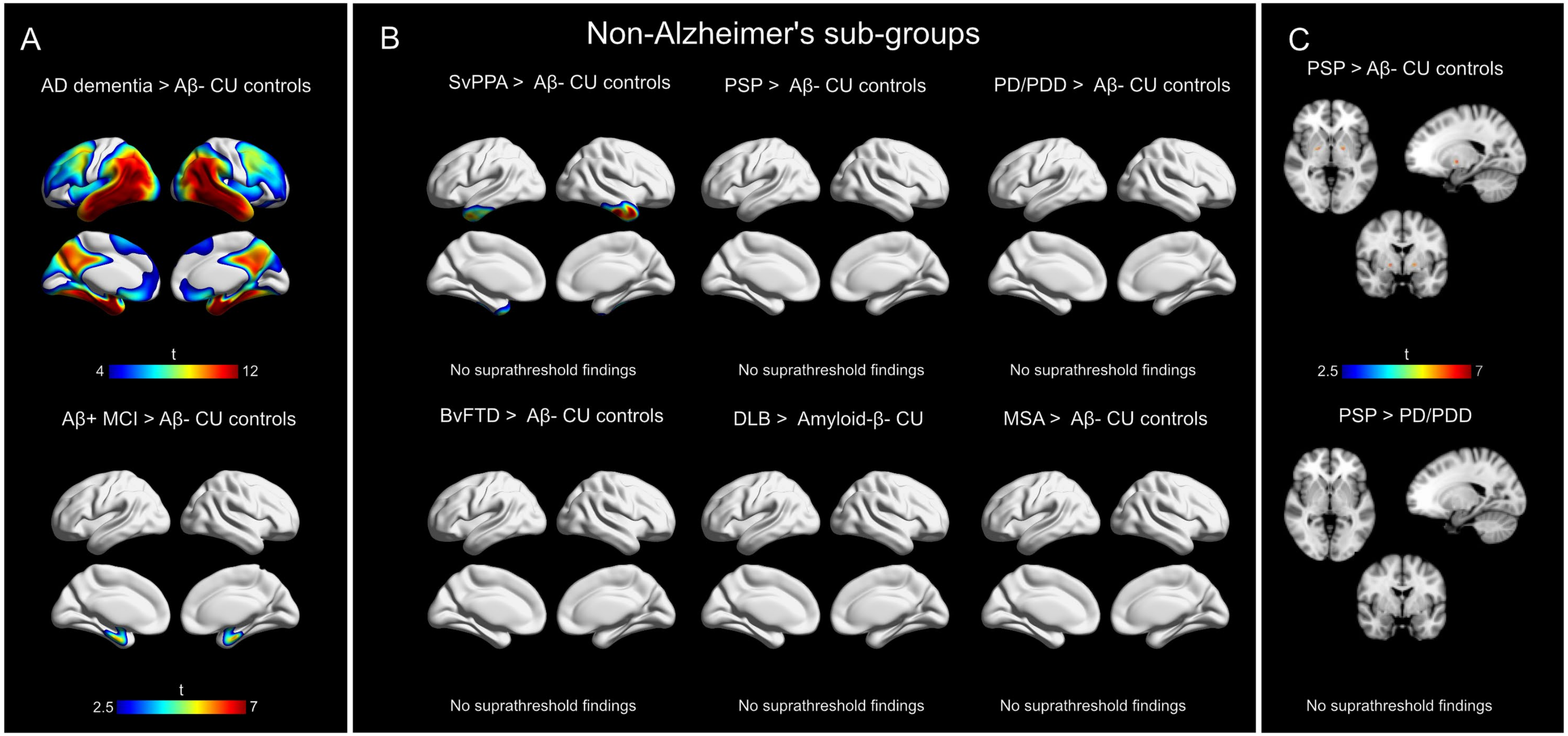


(A) Alzheimer's disease dementia greater than Aβ-negative cognitively unimpaired (CU) controls and Aβ-positive mild cognitive impairment. (B) Non-Alzheimer's disease sub-groups as compared Aβ-negative CU controls. (C) Progressive supranuclear palsy greater than Aβ-negative CU controls and Parkinson's disease. Parametric maps were adjusted for multiple comparisons using family wise error correction ($p < 0.05$, cluster extent $\geq 100$ voxels).

**eFigure 5.** Partial volume corrected [$^{18}$F]RO948 standardized uptake values ratios (SUVRs) across diagnostic groups within tau-imaging ROIs

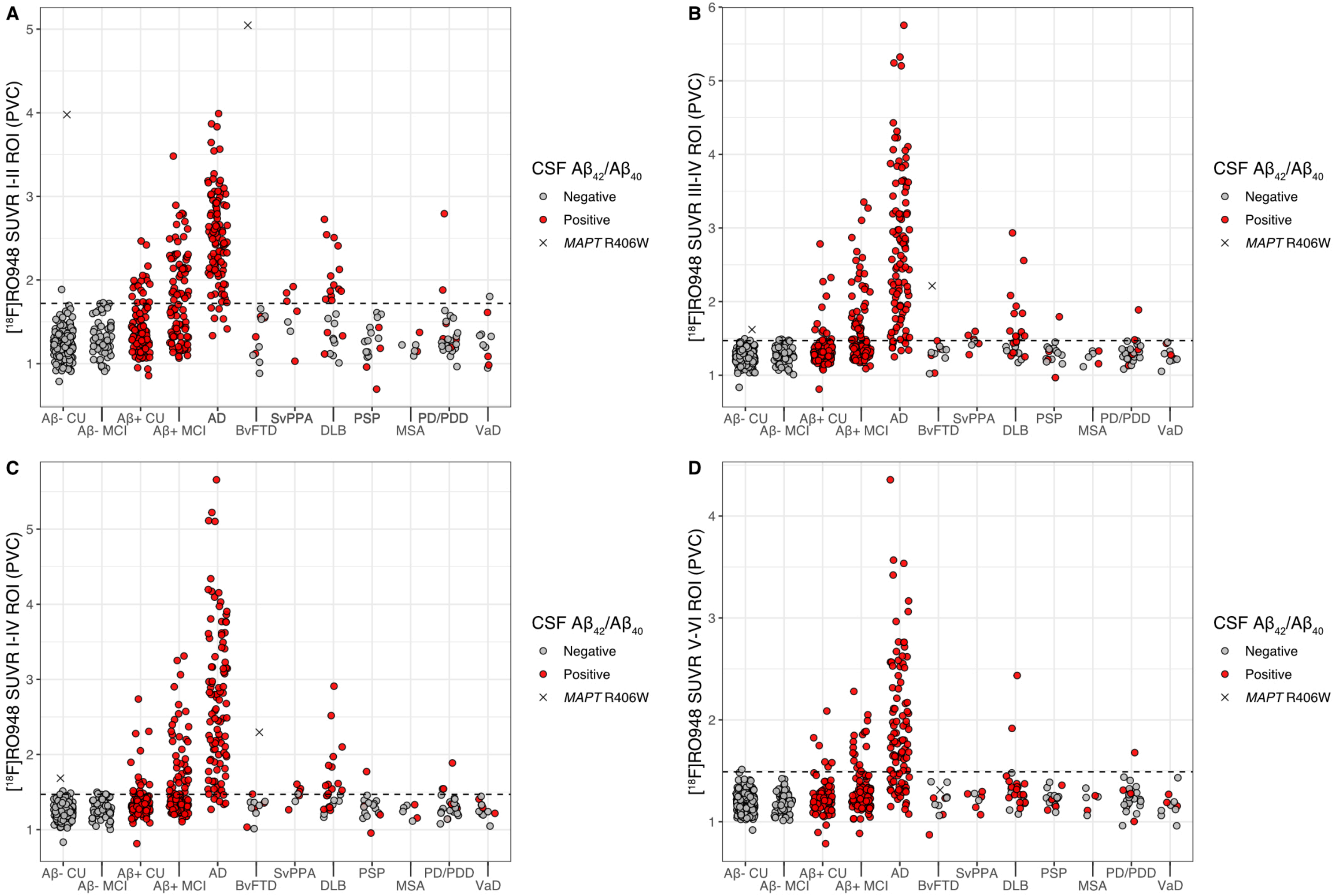


© 2020 Leuzy A et al. *JAMA Neurology*. 

Dashed lines indicate cutoffs (mean+2.5SD) derived from young Aβ-negative controls using partial volume error corrected [$^{18}$F]RO948 SUVR data: 1.72 (tau imaging I-II ROI), 1.47 (tau-imaging III-IV I-IV ROIs) and 1.45 (tau imaging V-VI ROI). The cutoff used to define CSF amyloid-β was 0.089 using $Aβ_{42}/Aβ_{40}$. Aβ- CU, Aβ-negative cognitively unimpaired control; Aβ- MCI Aβ-negative mild cognitive impairment; Aβ+ CU, Aβ-positive cognitively unimpaired control; Aβ+ MCI Aβ-positive mild cognitive impairment; AD, Alzheimer's disease dementia; BvFTD, behavioural variant frontotemporal dementia; SvPPA, semantic variant primary progressive aphasia; DLB, dementia with Lewy bodies; PSP, progressive supranuclear palsy; MSA, multiple system atrophy; PD/PDD, Parkinson's disease with and without dementia; VaD, vascular dementia.

**eFigure 6.** Concordance plots between partial volume corrected [[18]F]RO948 standardized uptake values ratios (SUVRs) and CSF A$\beta_{42}$/A$\beta_{40}$

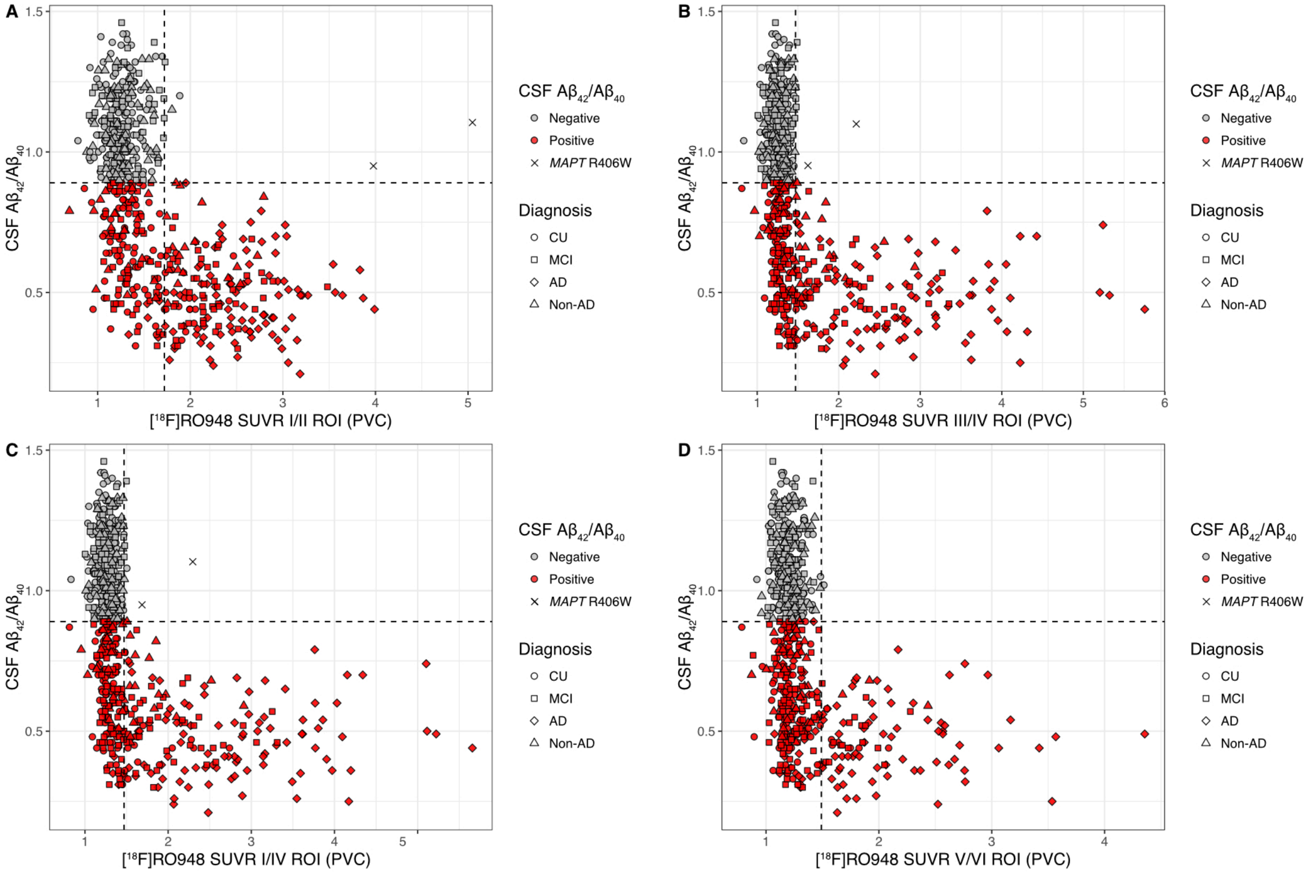


Plots A-D show concordance between [[18]F]RO948 SUVR and CSF A$\beta_{42}$/A$\beta_{40}$. The horizontal dashed lines indicate the cutoffs for tau-PET positivity across tau imaging stages, defined using the mean + 2.5 standard deviations in Aβ-negative young controls (I-II > 1.48; III-IV and I-IV ROIs > 1.36, V-VI ROI > 1.35). The vertical dashed line indicates the cutoff for Aβ-positivity (CSF A$\beta_{42}$/A$\beta_{40}$ < .089, as established by the neurochemistry laboratory at the Sahlgrenska University Hospital, Mölndal, Sweden).

**eFigure 7.** [18F]RO948 SUVR across tau-imaging ROIs using lower cutoffs

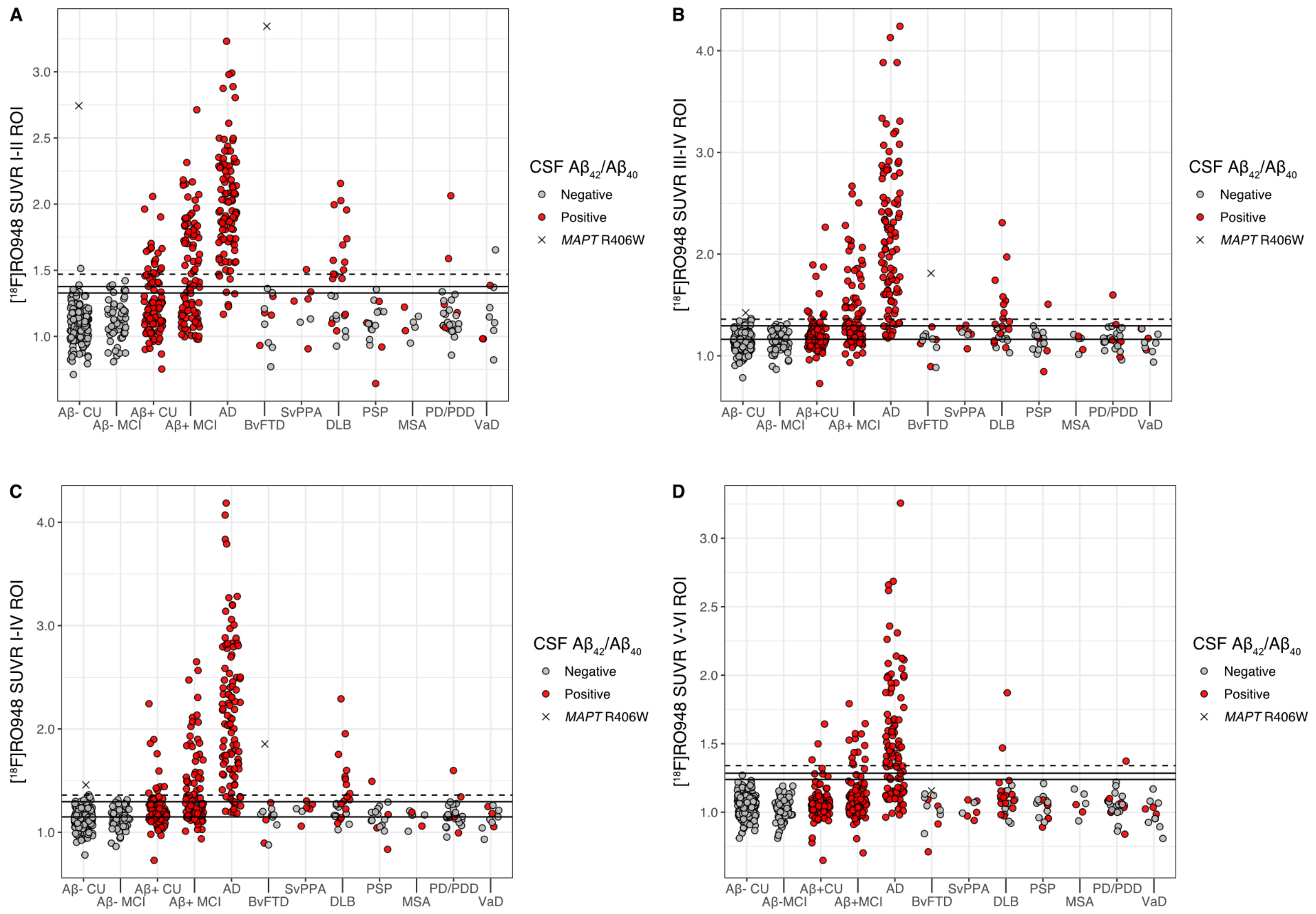

Dashed lines indicate cutoffs derived from Aβ-negative young-controls using the mean + 2.5 SD (I-II: 1.48; III-IV and I-IV, 1.36; V-VI, 1.35). The solid lines indicate cutoffs derived from Aβ-negative young-controls using the mean + 2 SD (I-II: 1.41; III-IV and I-IV, 1.28; V-VI, 1.30). and 1.5 SD (I-II: 1.32; III-IV and I-IV, 1.22; V-VI, 1.24). The cutoff used to define CSF amyloid-β was 0.089 using $Aβ_{42}/Aβ_{40}$. Aβ- CU, Aβ-negative cognitively unimpaired control; Aβ- MCI Aβ-negative mild cognitive impairment; Aβ+ CU, Aβ-positive cognitively unimpaired control; Aβ+ MCI Aβ-positive mild cognitive impairment; AD, Alzheimer's disease dementia; BvFTD, behavioural variant frontotemporal dementia; SvPPA, semantic variant primary progressive aphasia; DLB, dementia with Lewy bodies; PSP, progressive supranuclear palsy; MSA, multiple system atrophy; PD/PDD, Parkinson's disease with and without dementia; VaD, vascular dementia.

**eFigure 8.** [$^{18}$F]RO948 SUVR across tau-imaging ROIs by age (above and below 65)

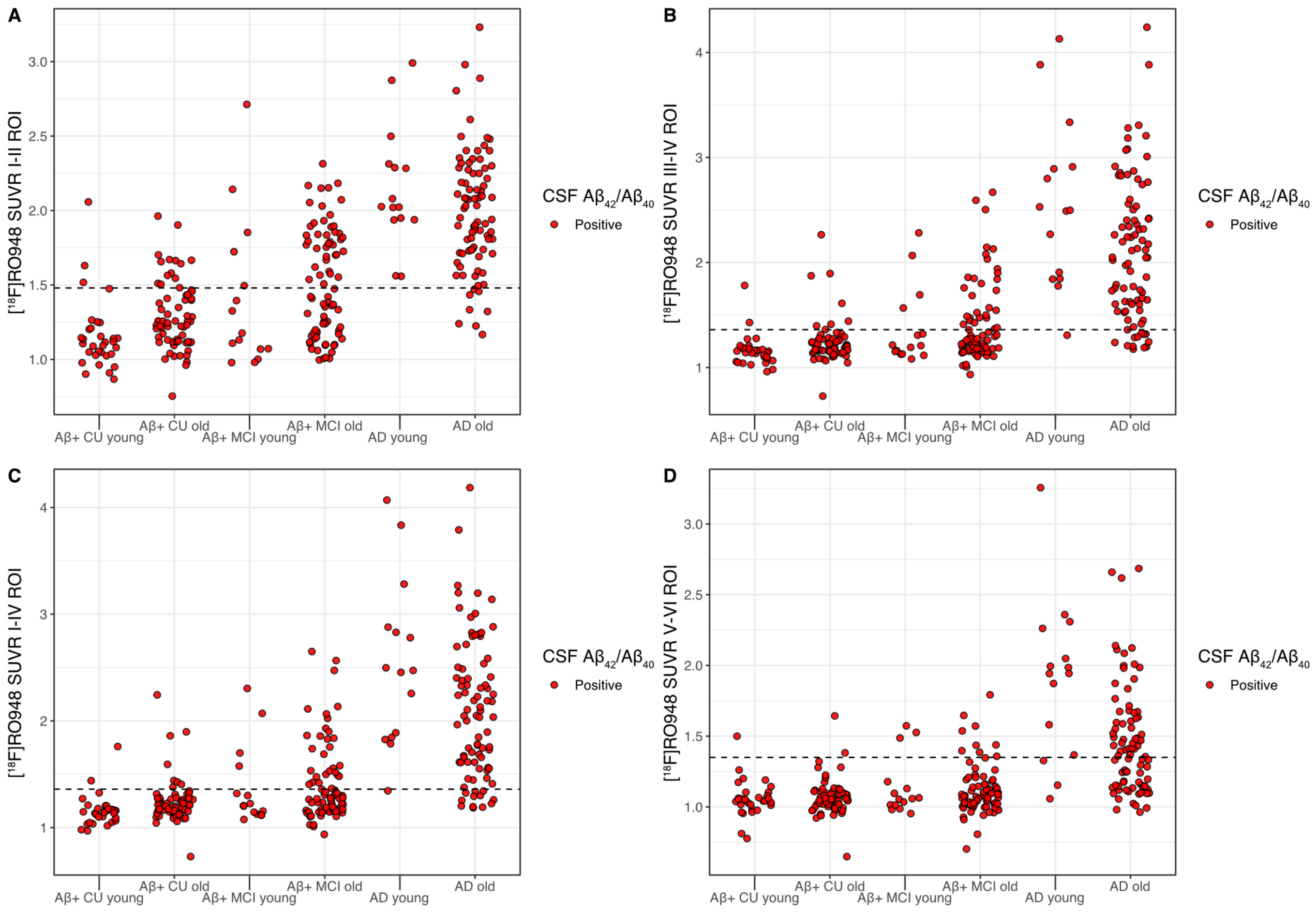

[$^{18}$F]RO948 SUVR across tau imaging stages are shown for Aβ-positive CU controls and MCI subjects, as well as patients with AD dementia, divided into young and old using age 65 as a cutoff. The horizontal dashed lines indicate the cutoffs for tau-positivity across tau-imaging ROIs, defined using the mean + 2.5 standard deviations in Aβ-negative young controls (I-II > 1.48; III-IV and I-IV ROIs > 1.36, V-VI ROI > 1.35). The cutoff used to defined CSF Aβ-positivity (CSF $A\beta_{42}/A\beta_{40}$ < .089) was as established by the neurochemistry laboratory at the Sahlgrenska University Hospital, Mölndal, Sweden. Aβ+ CU, Aβ-positive cognitively unimpaired control; Aβ+ MCI Aβ-positive mild cognitive impairment; AD, Alzheimer's disease dementia.

**eFigure 9.** [$^{18}$F]RO948 SUVR in primary somatosensory and motor cortices

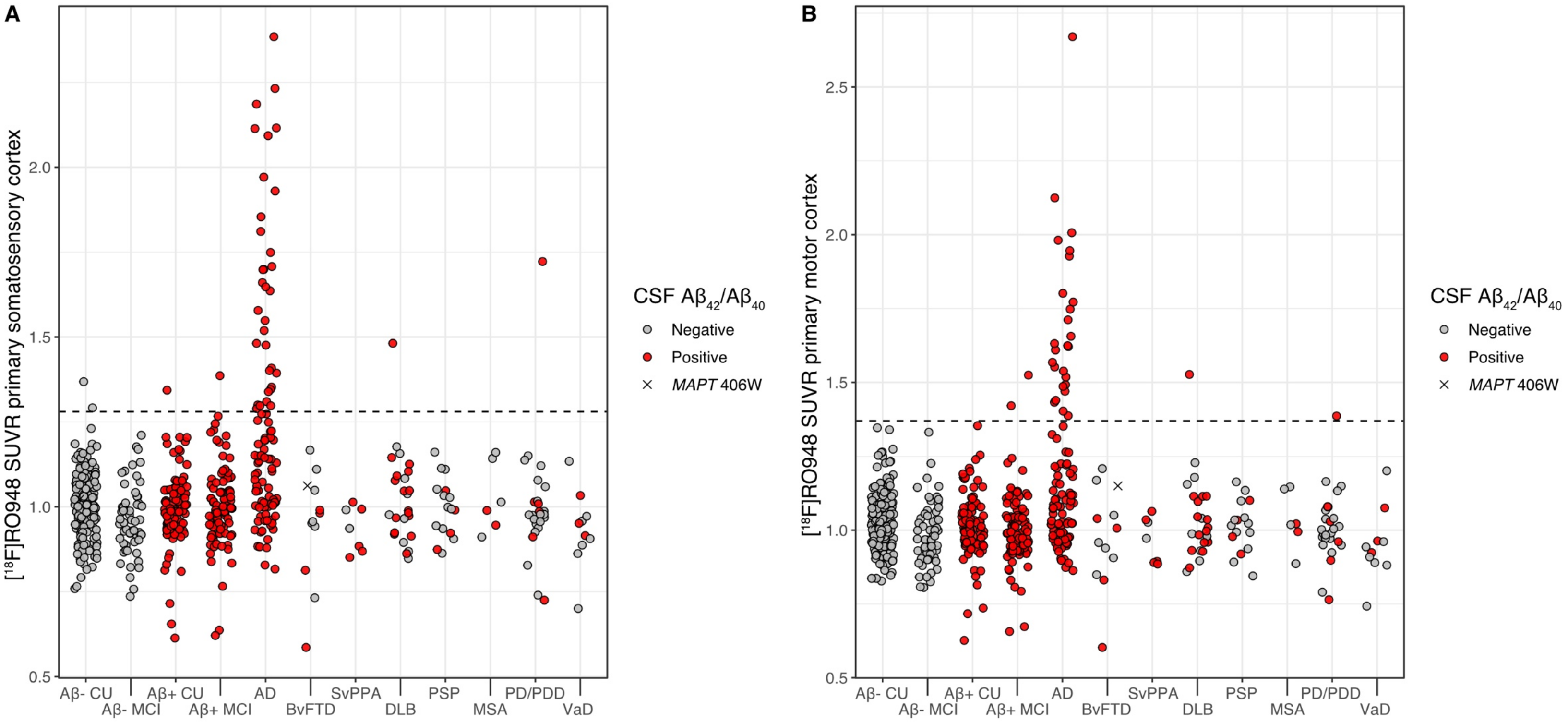


© 2020 Leuzy A et al. *JAMA Neurology*. 

Plots showing [$^{18}$F]RO948 SUVR in primary somatosensory (A) and motor cortices (B). The horizontal dashed lines indicate the cutoffs for tau-positivity, defined using the mean + 2.5 standard deviations in Aβ-negative young controls. The cutoff for Aβ-positivity (CSF $A\beta_{42}/A\beta_{40} < .089$, as established by the neurochemistry laboratory at the Sahlgrenska University Hospital, Mölndal, Sweden). Aβ- CU, Aβ-negative cognitively unimpaired control; Aβ- MCI, Aβ-negative mild cognitive impairment; Aβ+ CU, Aβ-positive cognitively unimpaired control; Aβ+ MCI Aβ-positive mild cognitive impairment; AD, Alzheimer's disease dementia; BvFTD, behavioural variant frontotemporal dementia; SvPPA, semantic variant primary progressive aphasia; DLB, dementia with Lewy bodies; PSP, progressive supranuclear palsy; MSA, multiple system atrophy; PD/PDD, Parkinson's disease with and without dementia; VaD, vascular dementia.

**eFigure 10.** Mean [$^{18}$F]RO948 standardized uptake values ratios (SUVR) across diagnostic groups (DLB subdivided by Aβ-status) within tau-imaging ROIs

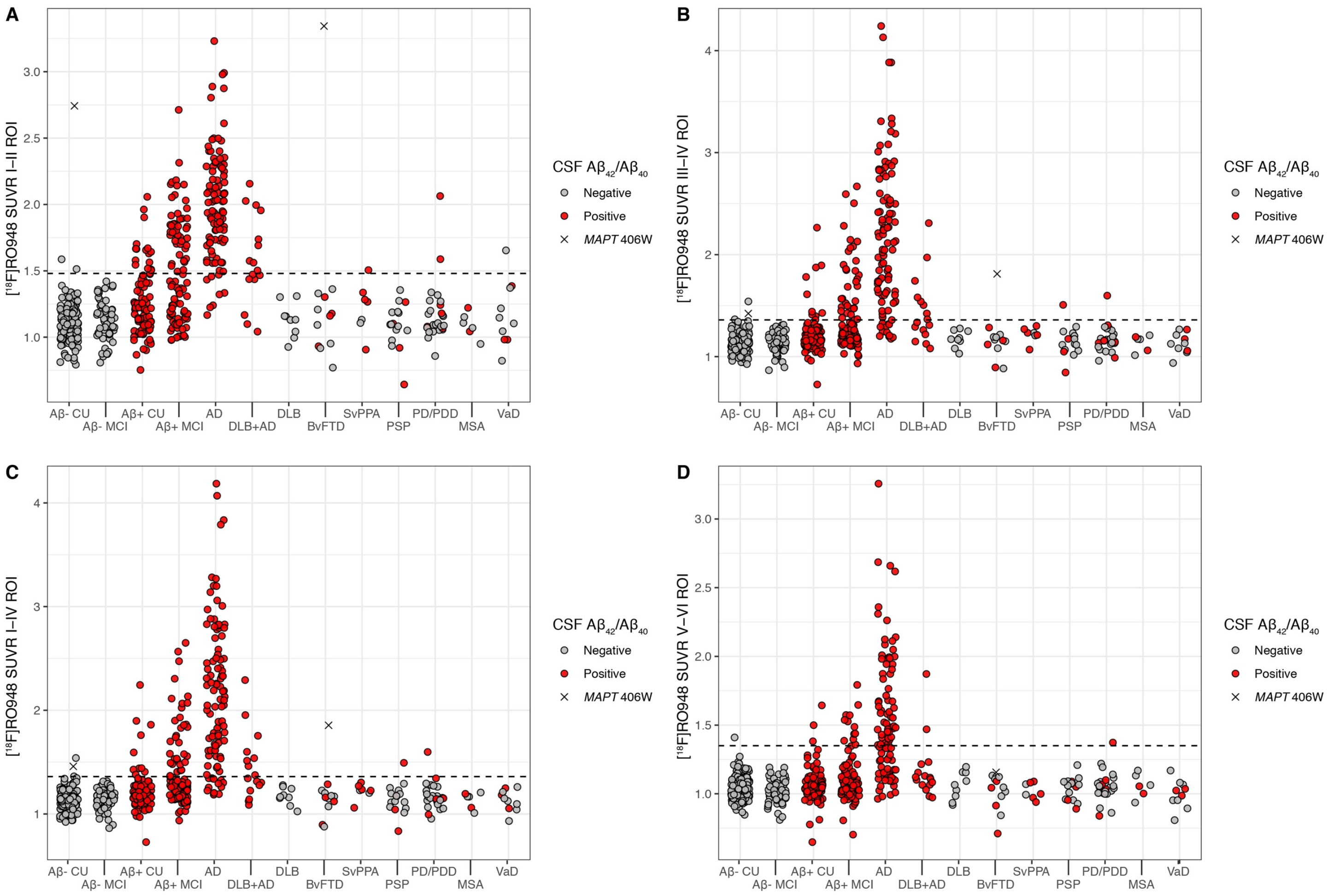

The horizontal dashed lines indicate the cutoffs for tau-positivity across tau-imaging ROIs, defined using the mean + 2.5 standard deviations in Aβ-negative young controls (I-II > 1.48; III-IV and I-IV ROIs > 1.36, V-VI ROI > 1.35).

**eFigure 11.** Plots from receiver operating characteristic analyses ([$^{18}$F]RO948, MRI- and CSF-measures) for distinguishing AD dementia (A) and Aβ-positive MCI (B) from non-AD neurodegenerative disorders (Aβ+ DLB [DLB+AD] excluded, n=16)

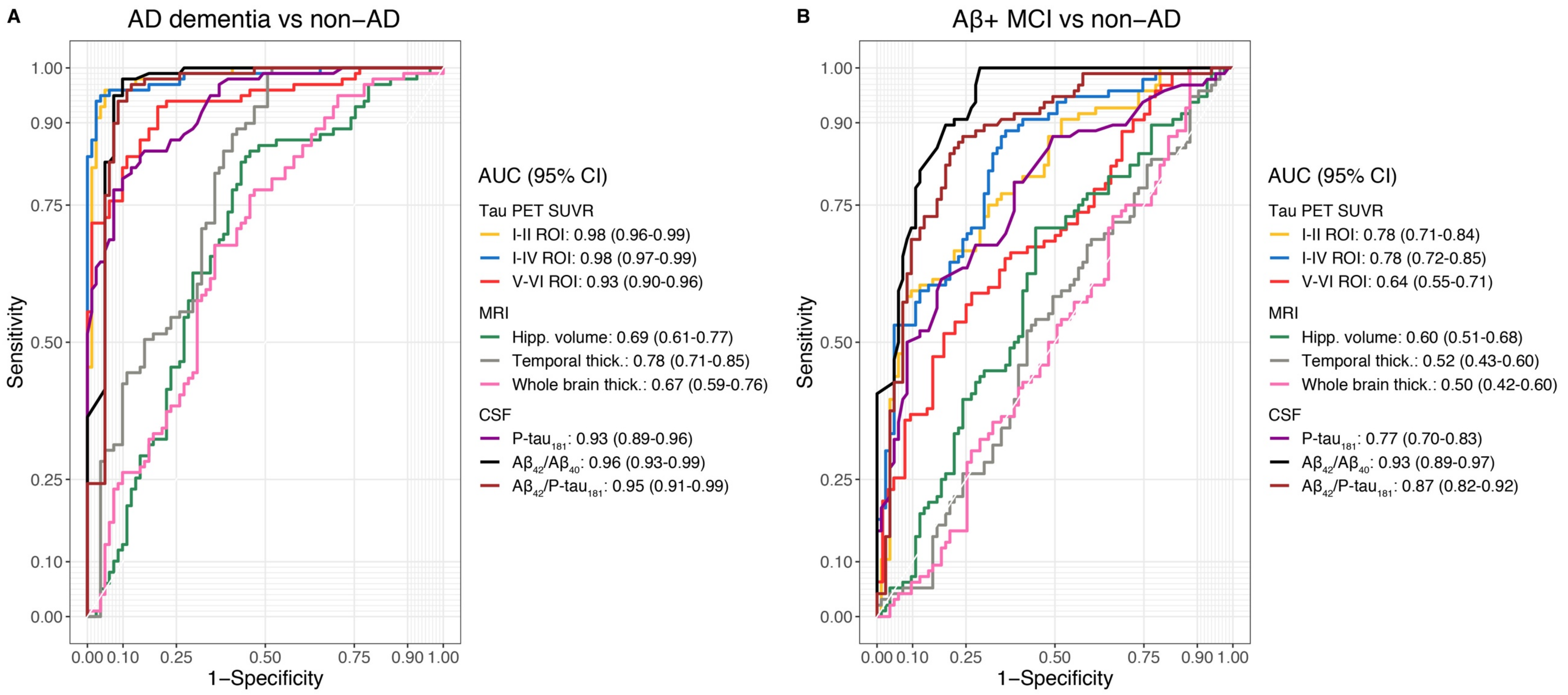

**eFigure 12.** CSF P-tau181 levels by diagnostic group

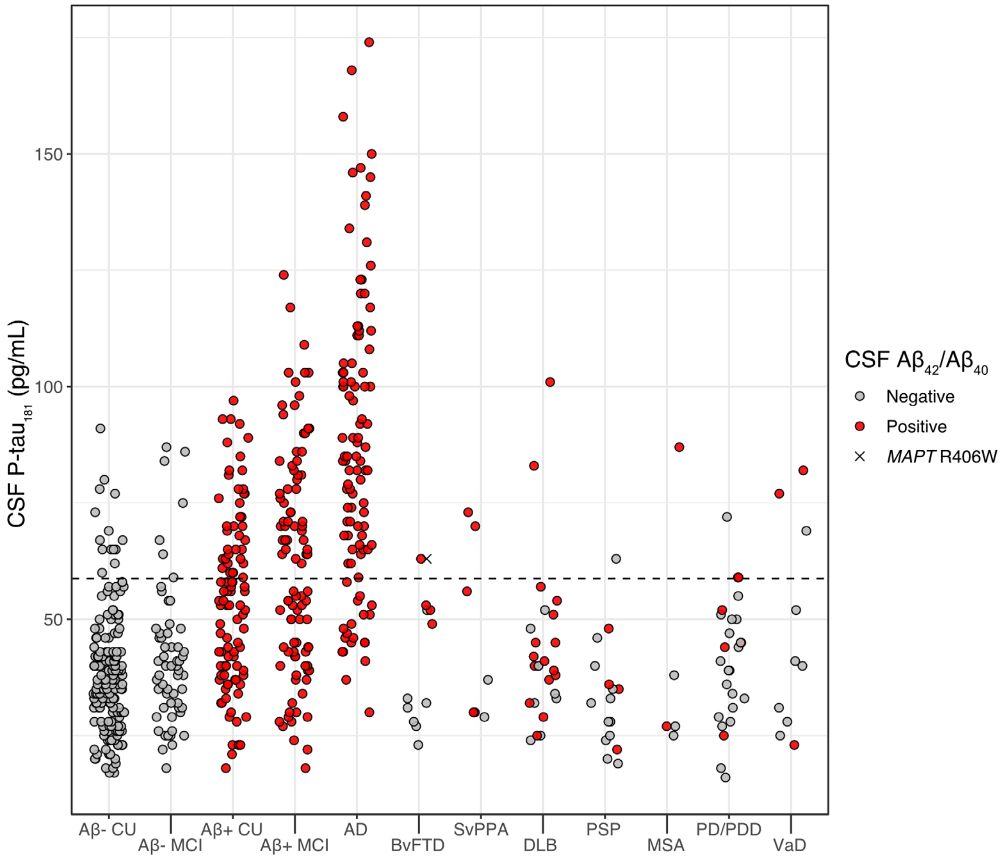


Aβ- CU, Aβ-negative cognitively unimpaired control; Aβ- MCI Aβ-negative mild cognitive impairment; Aβ+ CU, Aβ-positive cognitively unimpaired control; Aβ+ MCI Aβ-positive mild cognitive impairment; AD, Alzheimer's disease dementia; BvFTD, behavioural variant frontotemporal dementia; SvPPA, semantic variant primary progressive aphasia; DLB, dementia with Lewy bodies; PSP, progressive supranuclear palsy; MSA, multiple system atrophy; PD/PDD, Parkinson's disease with and without dementia; VaD, vascular dementia. The dashed line indicates the cutoff of 60.79 pg/mL, set in BioFINDER-2 cohort using Gaussian mixture modelling (n=850).

**eFigure 13.** CSF P-tau181 levels across tau-imaging ROIs

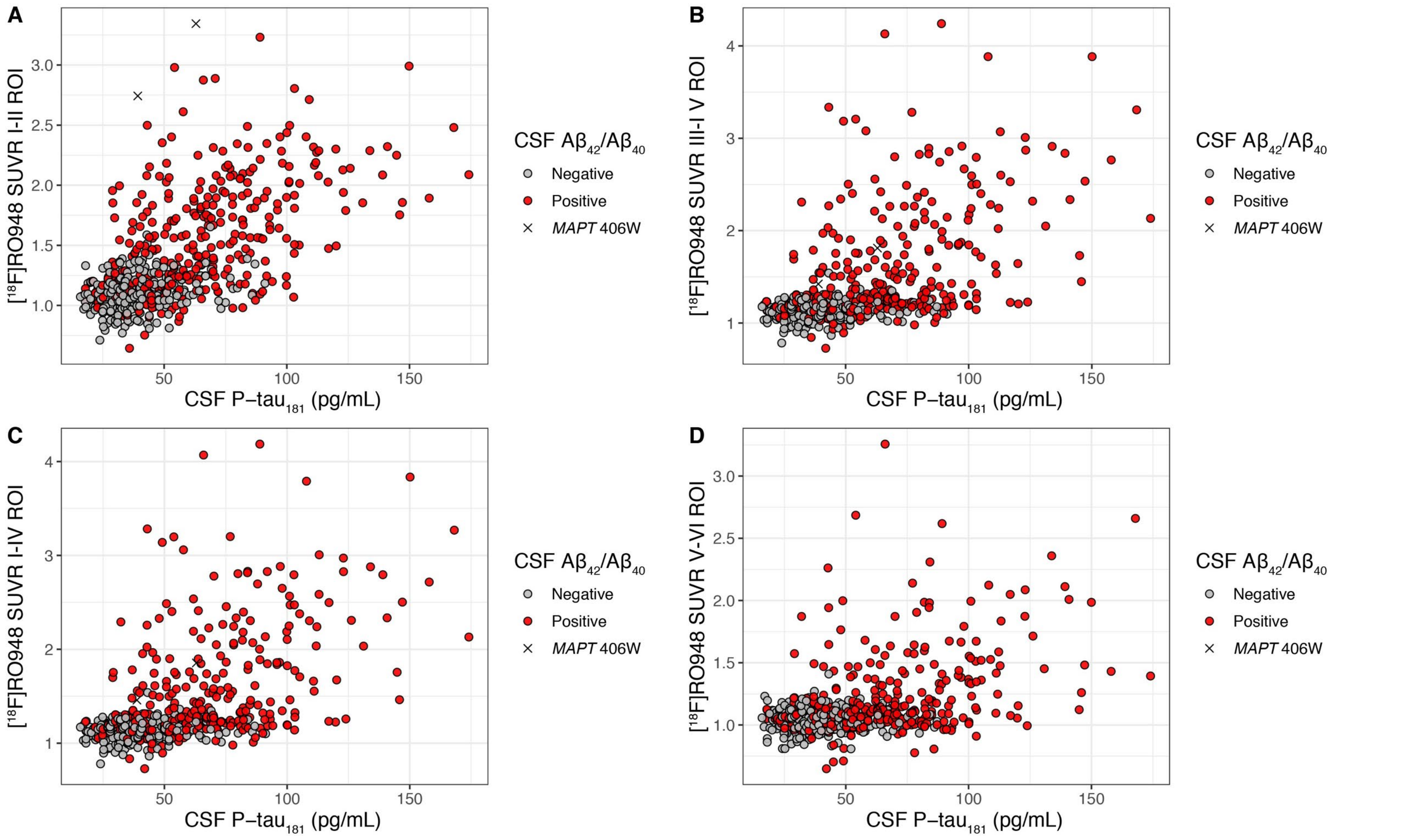


© 2020 Leuzy A et al. *JAMA Neurology*. 

CSF P-$tau_{181}$was significantly correlated with [$^{18}$F]RO948 PET SUVR in tau-imaging ROIs: I-II (r=0.62, *P*<.001, III-IV (r=0.57, *P*<.001), I-IV (r=0.58, *P*<.001) and V-VI (r=0.46, *P*<.001).

**eFigure 14.** [$^{18}$F]RO948 and [$^{18}$F]flortaucipir PET in semantic variant primary progressive aphasia

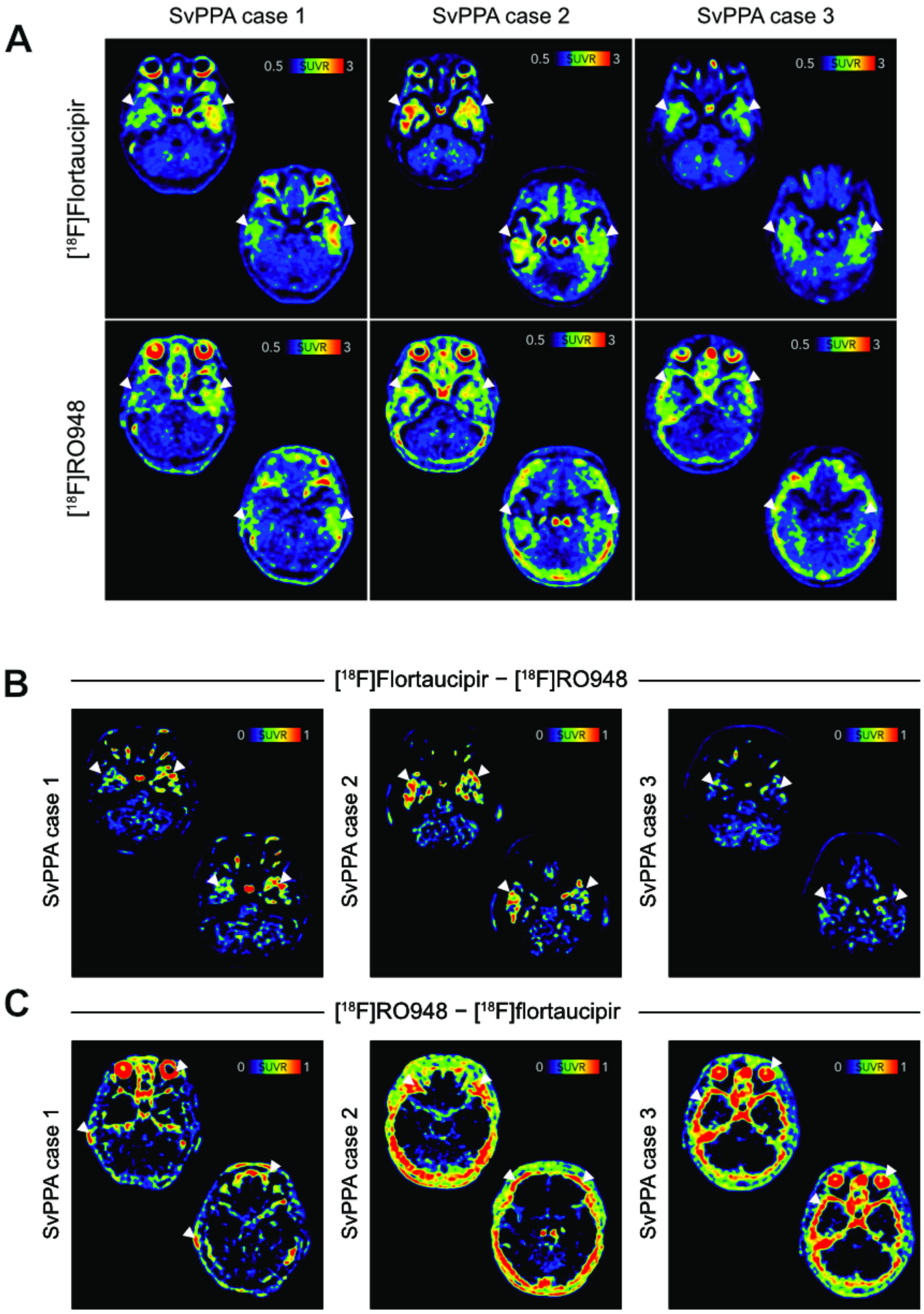


(A) [$^{18}$F]flortaucipir and [$^{18}$F]RO948 SUVR PET images of three cases. The inverted white triangles indicate the anterior temporal lobes, where signal was seen with [$^{18}$F]flortaucipir

(top row) and [$^{18}$F]RO948 (bottom row). Subtraction images of the three cases are show in (B) and (C). In (B), [$^{18}$F]Flortaucipir - [$^{18}$F]RO948, with the inverted white triangles indicating areas where [$^{18}$F]flortaucipir SUVR was higher than [$^{18}$F]RO948 SUVR. In (C), [$^{18}$F]RO948 -[$^{18}$F]flortaucipir is shown, with the inverted white triangles indicating areas where [$^{18}$F]RO948 SUVR was higher than [$^{18}$F]flortaucipir SUVR.

**eFigure 15.** Decision tree outlining the potential clinical utility of tau-PET imaging across different dementia disorders, including Alzheimer disease

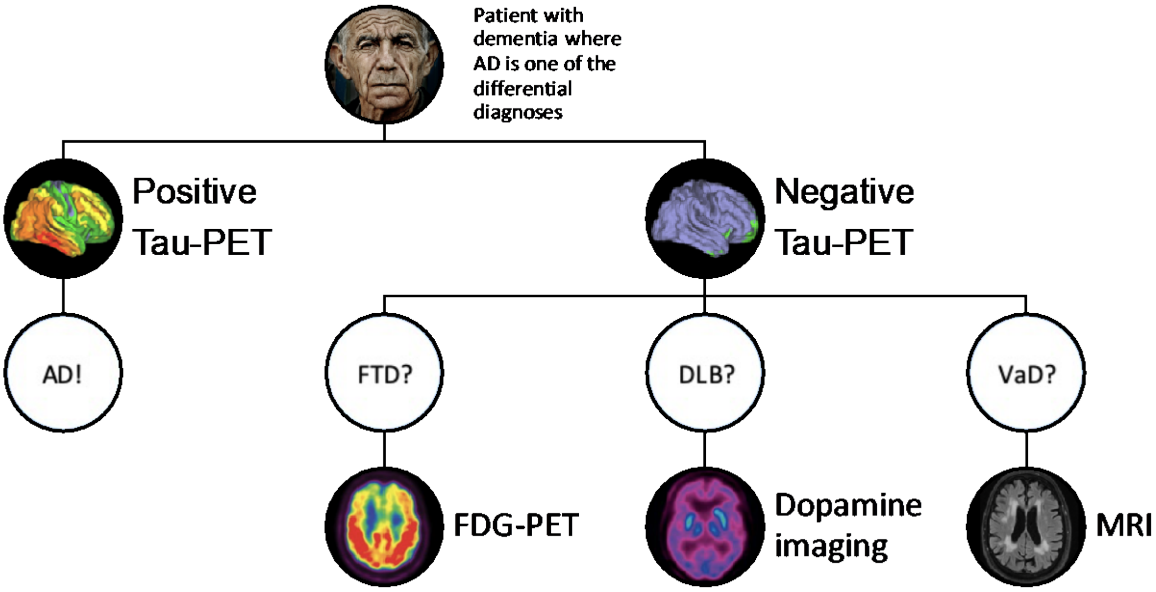


In the event of a positive (Pos.) tau scan, a diagnosis of Alzheimer's disease (AD) would be most likely. In the event of a negative (Neg.) scan, additional imaging based investigations would be ordered on the basis of the primary suspected differential diagnosis. In the case of a frontotemporal dementia (FTD) disorder (i.e. behavioural variant FTD and semantic variant primary progressive aphasia), metabolic imaging with [$^{18}$F]FDG would be used. In the event of dementia with Lewy bodies (DLB) being suspected, dopamine transporter (DAT) imaging would be ordered (PET or SPECT based) and, possibly, [$^{18}$F]FDG. In the case of vascular dementia (VaD), MRI with suitable sequences would be done.
